# Supplementary material for: Intracellular interleukin-32γ mediates antiviral activity of cytokines against hepatitis B virus
Source: Nat Commun. 2018 Aug 16;9:3284. doi: 10.1038/s41467-018-05782-5 (PMC6095909; doi:10.1038/s41467-018-05782-5)
Supplement: Supplementary file 1 — Supplementary Information [file 41467_2018_5782_MOESM1_ESM.pdf]

Supplementary Information

Kim *et al*

Intracellular interleukin-32γ mediates the antiviral activity of cytokines against Hepatitis B virus

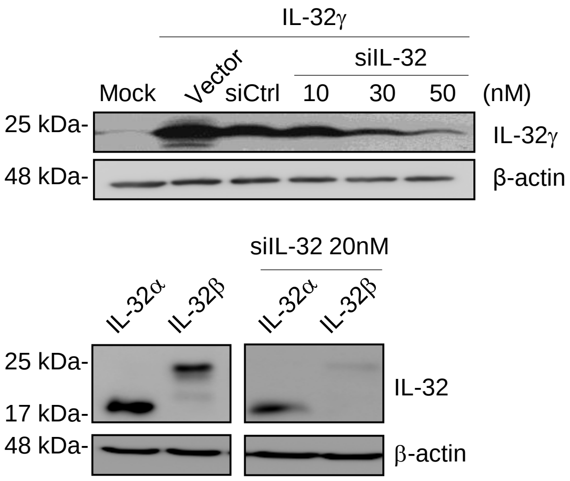

Supplementary Figure 1. Silencing effect of IL-32 siRNA on IL-32 isoforms. Expression plasmids for IL-32 isoforms (IL-32α, IL-32β, and IL-32γ) were transfected into Huh7 cells and the silencing effect was evaluated at 48 h post-transfection by Western blotting.

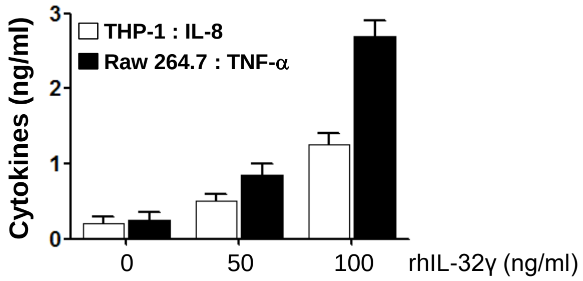

Supplementary Figure 2. Recombinant human IL-32γ (rhIL-32γ) is functionally active. THP-1 and Raw 264.7 cells were treated with rhIL-32γ (50 or 100 ng/ml) for 18 h, and the culture supernatants were used for cytokine measurement. Human IL-8 secreted from THP-1 cells and mouse TNF-α secreted from Raw 264.7 cells were measured by ELISA. Values were obtained from three independent experiments (means ± SD).

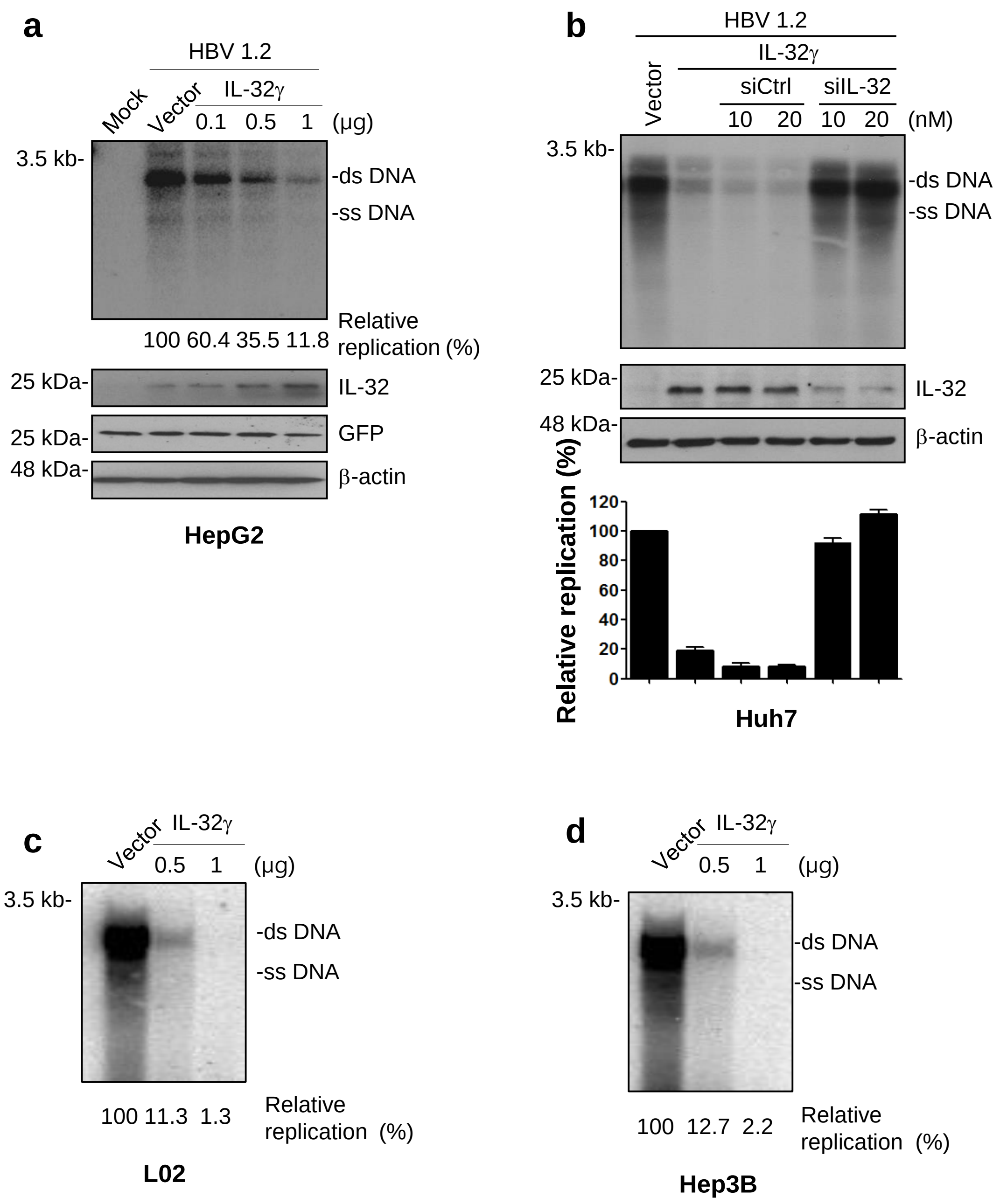

Supplementary Figure 3. IL-32γ strongly suppresses HBV replication in HepG2, Huh7, L02, and Hep3B cells. a Dose-dependent suppression of HBV replication by IL-32γ expression in HepG2 cells. The levels of HBV replication and protein expression were analyzed by Southern and Western blotting, respectively. **b** Rescue of viral replication by IL-32γ knock-down in Huh7 cells. **c, d** Dose-dependent suppression of HBV replication by IL-32γ expression in L02 and Hep3B cells. Data were obtained from three independent experiments (mean ± S.D.).

a

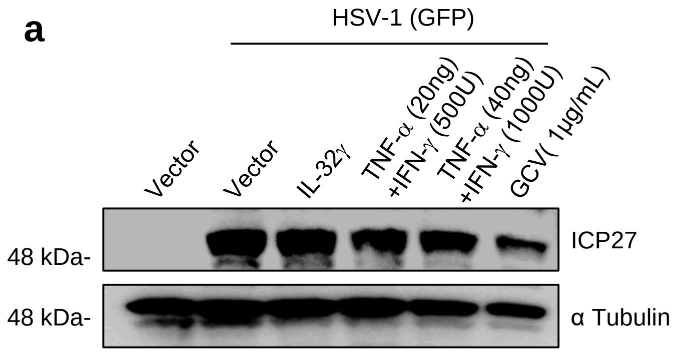

b

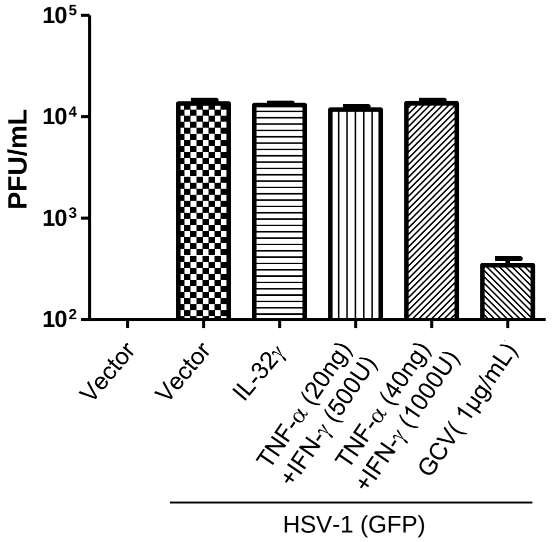

Supplementary Figure 4. No suppressive effect of IL-32γ on herpes simplex virus 1 (HSV-1). IL-32 expression vector or empty vector was transfected into Huh7 cells. After 16 h, cells were infected with HSV-1_GFP at 1 MOI. Cell treatment with GCV (1 μg/mL) was used as a positive control. **a** The level of viral ICP27 protein was analyzed at 24 h post-infection by Western blotting. **b** Viral titers were analyzed by plaque assay with Vero cells. Data were obtained from three independent experiments (mean ± S.D.).

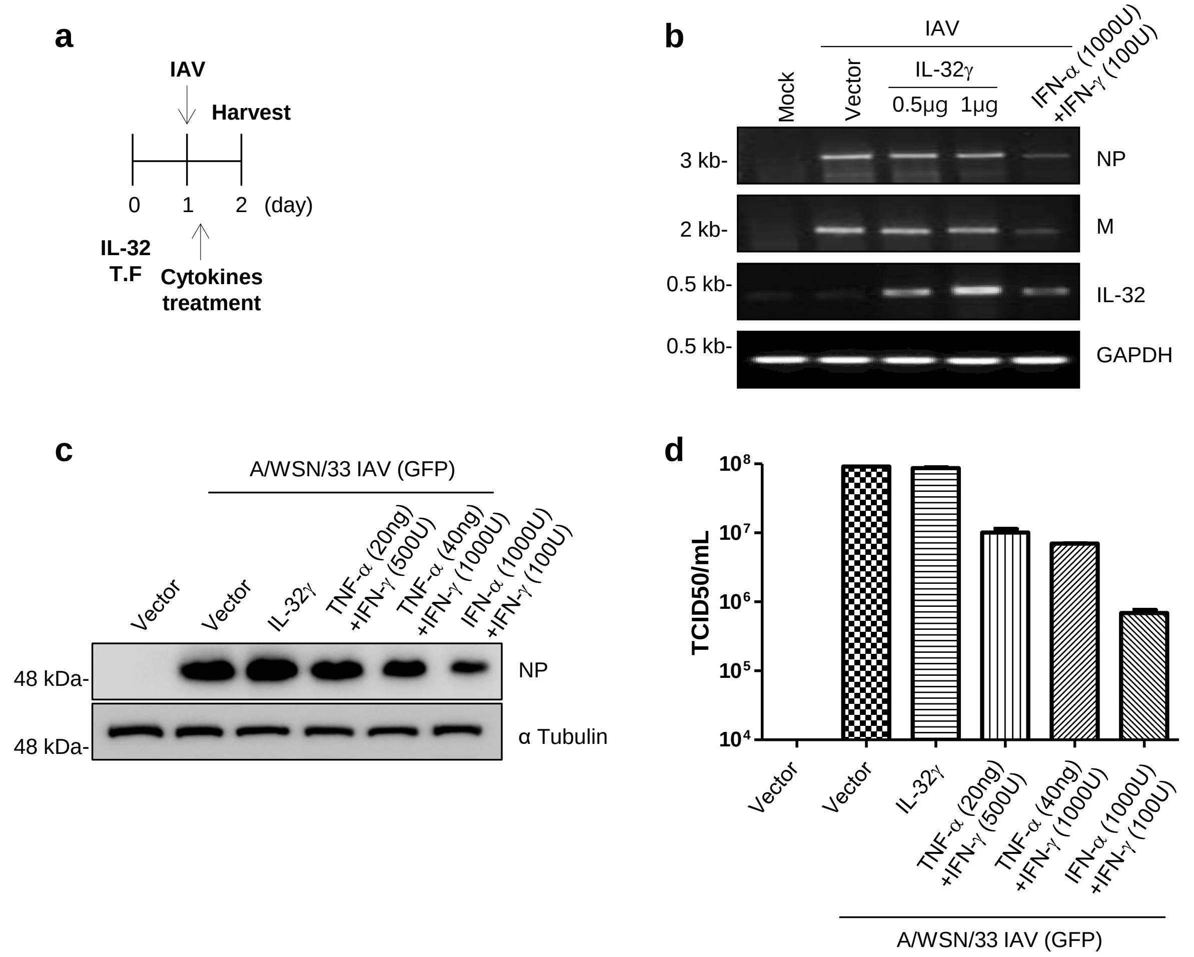

Supplementary Figure 5. No suppressive effect of IL-32γ on influenza virus. IL-32 expression vector or empty vector was transfected into Huh7 cells. After 16 h, cells were infected with A/WSN/33_GFP at 1 MOI. Cells treated with IFNs (1000 U/mL IFN-α and 100 U/mL IFN-γ) were used as a positive control. **a** The experimental scheme. **b** The levels of IL-32γ and viral NP and M RNAs were determined by semi-quantitative RT-PCR. **c** The level of viral NP protein was analyzed by Western blotting. **d** Viral titers were analyzed by TCID50 assay with MDCK cells. Data were obtained from three independent experiments (mean ± S.D.).

a

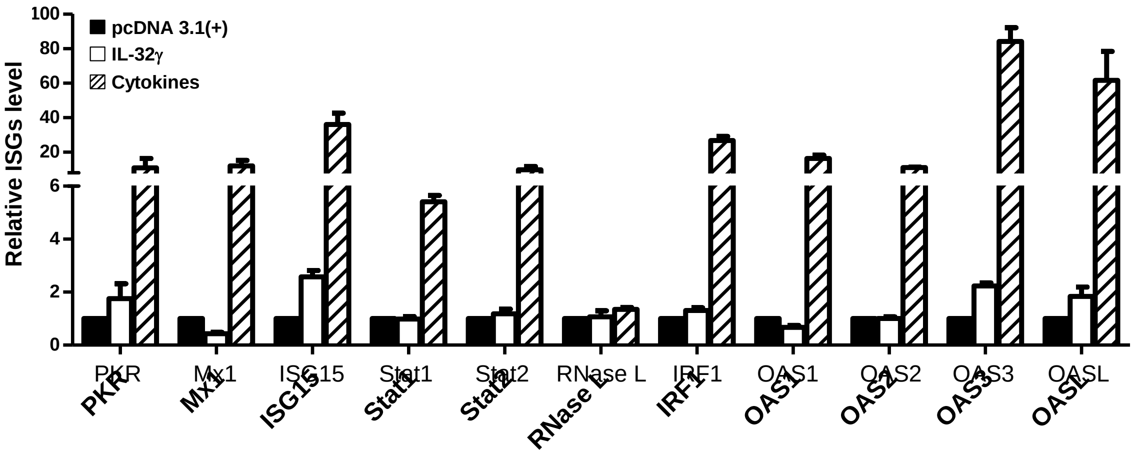

b

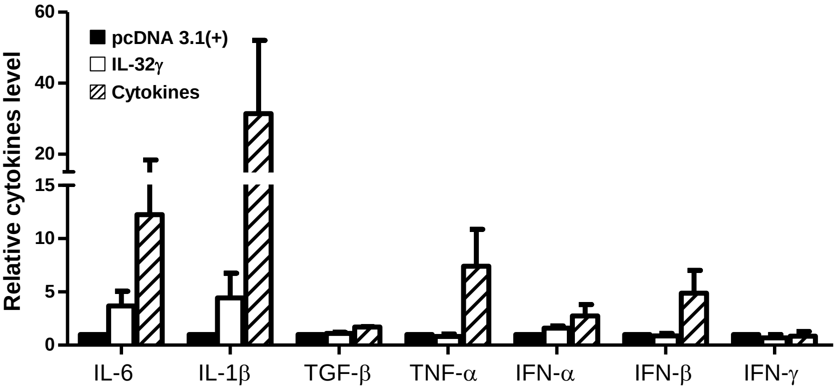

Supplementary Figure 6. Effect of IL-32γ on expression of interferon-stimulated genes (ISGs) and genes for other cytokines in PHHs. PHHs were either transfected or treated with IL-32γ or cytokines and quantitative real-time PCR was performed to analyze the expression levels of several ISGs. Cytokines (TNF-α and IFN-γ) were added for 48 h and total cDNA library was synthesized. Results are presented as the ratio to control transfection after amplification of each ISG. Data were obtained from triplicate experiments (mean ± S.D.). For each experiment, the relative fold values were normalized by the GAPDH expression level. **a**, **b** Effect of IL-32γ on the expression of representative cytokines **a** and ISGs **b**.

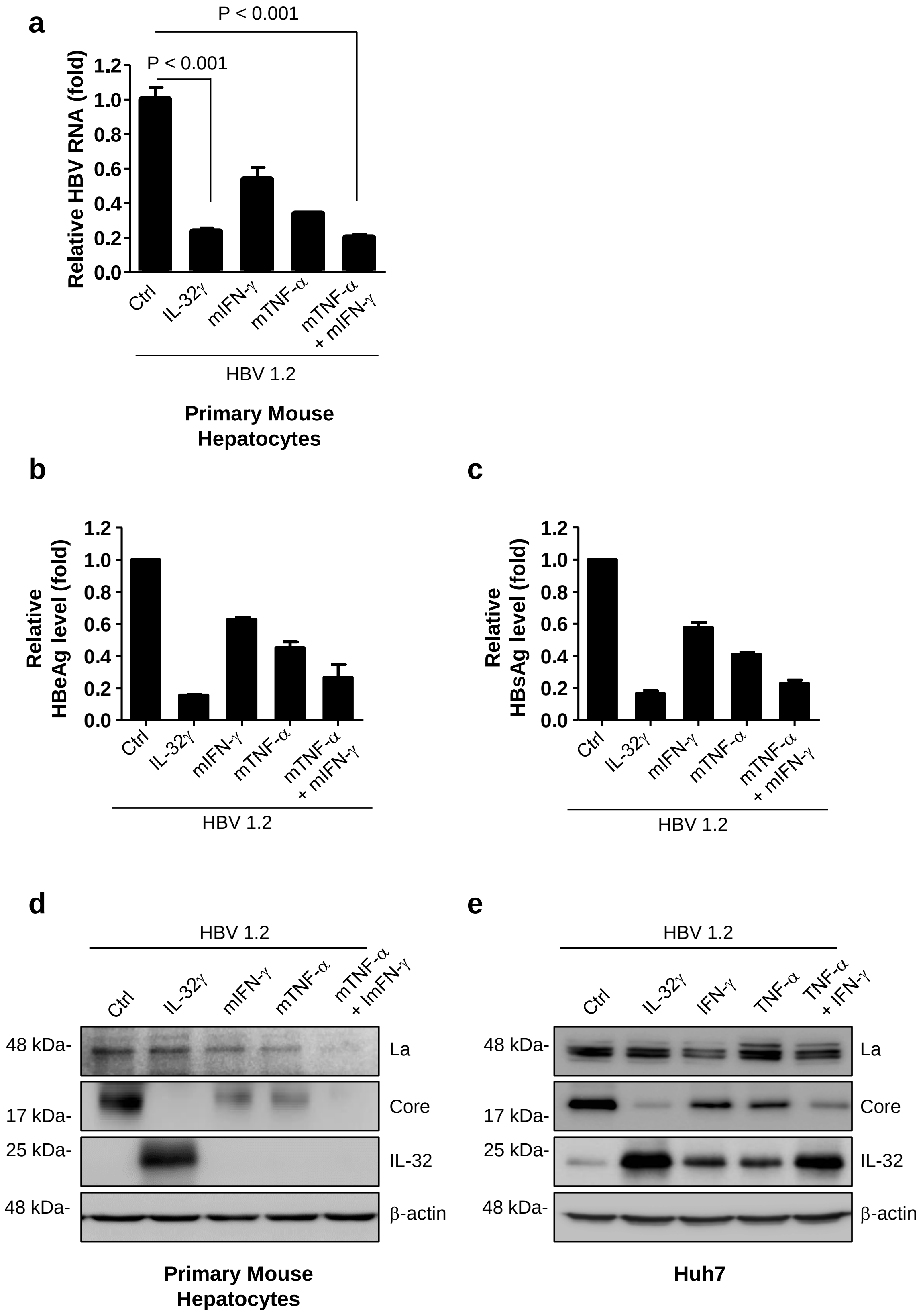
a
P < 0.001
P < 0.001
Relative HBV RNA (fold)
1.2
1.0
0.8
0.6
0.4
0.2
0.0
Ctrl
IL-32γ
mIFN-γ
mTNF-α
mTNF-α + mIFN-γ
HBV 1.2
Primary Mouse Hepatocytes
b
Relative HBeAg level (fold)
1.2
1.0
0.8
0.6
0.4
0.2
0.0
Ctrl
IL-32γ
mIFN-γ
mTNF-α
mTNF-α + mIFN-γ
HBV 1.2
c
Relative HBsAg level (fold)
1.2
1.0
0.8
0.6
0.4
0.2
0.0
Ctrl
IL-32γ
mIFN-γ
mTNF-α
mTNF-α + mIFN-γ
HBV 1.2
d
HBV 1.2
Ctrl
IL-32γ
mIFN-γ
mTNF-α
mTNF-α + lmFN-γ
48 kDa-
La
17 kDa-
Core
25 kDa-
IL-32
48 kDa-
β-actin
Primary Mouse Hepatocytes
e
HBV 1.2
Ctrl
IL-32γ
IFN-γ
TNF-α
TNF-α + IFN-γ
48 kDa-
La
17 kDa-
Core
25 kDa-
IL-32
48 kDa-
β-actin
Huh7

Supplementary Figure 7. IL-32γ and cytokines strongly suppress HBV mRNA in primary mouse hepatocytes. PMHs were isolated from six-week-old male mice. Expression plasmids for HBV 1.2 (1 μg) and IL-32 (1 μg) were transfected into PMHs using JetPEI Hepatocyte reagent. In parallel, PMHs were treated with mTNF-α (20 ng/mL) and mIFN-γ (500 U/mL). Human Huh7 cells were treated with human TNF-α (20 ng/ml) and IFN-γ (500 U/mL). After transfection with HBV 1.2, cytokines were added for 3 days in fresh medium. **a** HBV mRNA was determined by real-time PCR. **b**, **c** At 3 days post-transfection, the HBeAg and HBsAg levels were determined by ELISA. **d, e** The protein levels of La, HBV core, IL-32, and β-actin were determined by Western blotting in PMHs or Huh7 cells. Data were obtained from three independent experiments (mean ± S.D.). $p < 0.001$ by Student’s t-test.

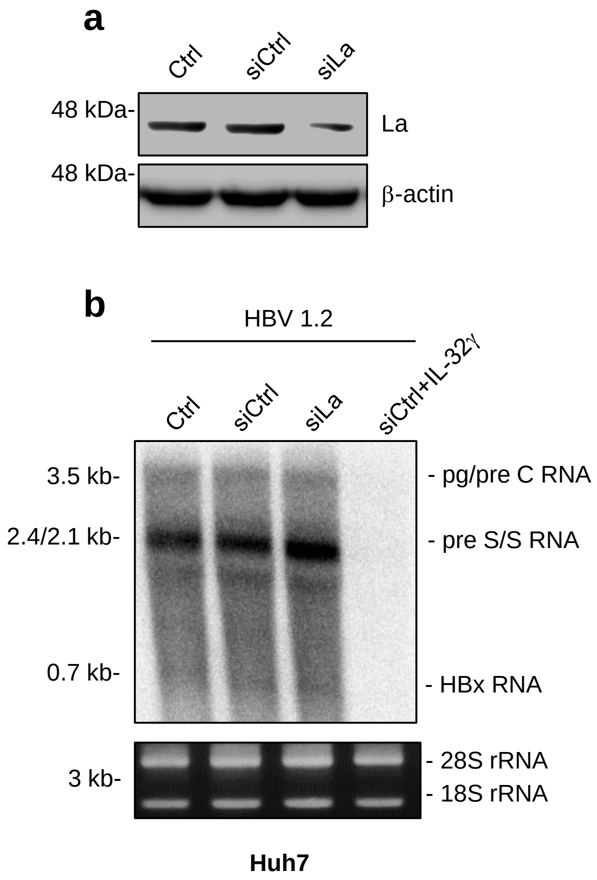

Supplementary Figure 8. Effect of SSB/La knock-down on the levels of HBV RNAs in Huh7 cells. HBV 1.2 (1 μg), siRNAs (20 nM), and/or IL-32γ (1 μg) plasmids were co-transfected into Huh7 cells with Lipofectamine 2000. **a** Validation of La knock-down by siRNA. Protein level was detected by Western blotting. **b** At 72 h post-co-transfection, the levels of HBV RNAs in Huh7 cells were determined by Northern blotting.

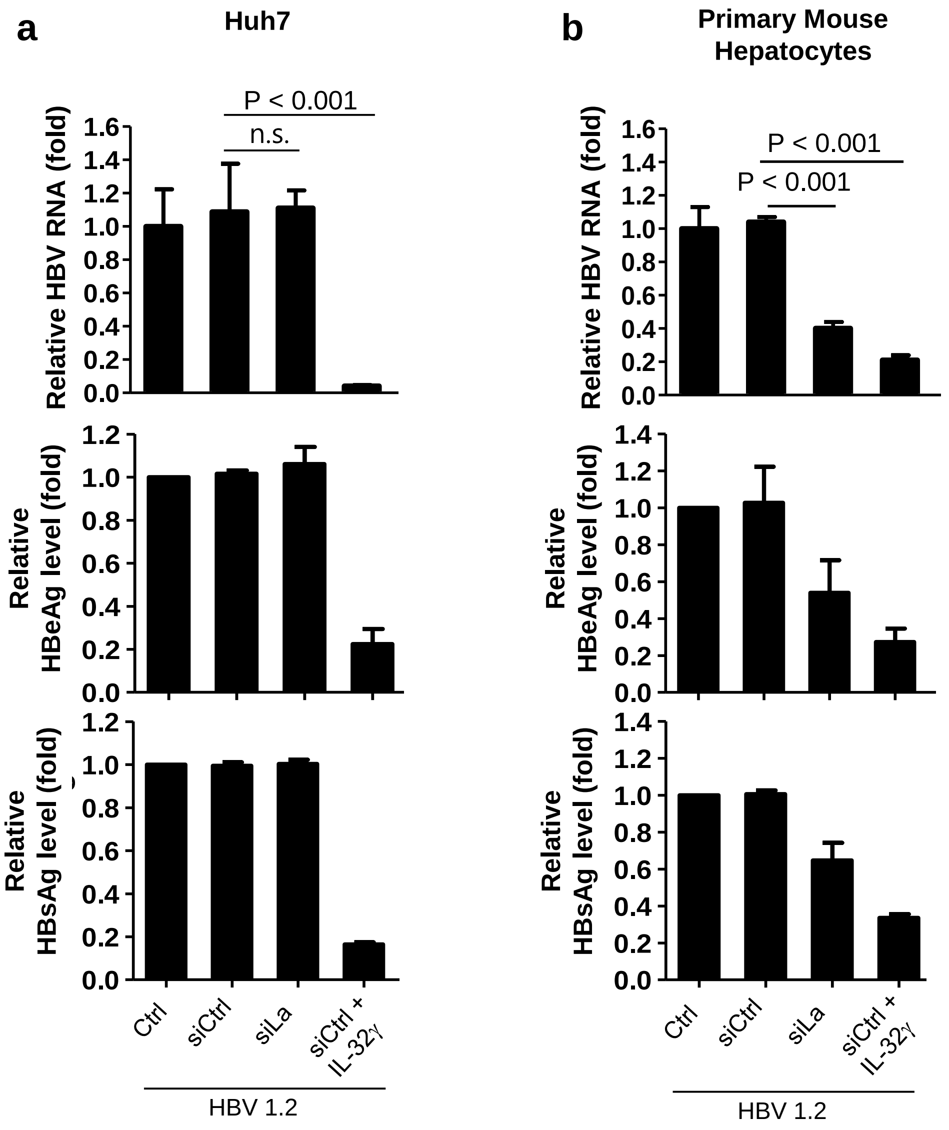

Supplementary Figure 9. Effect of La knock-down on the levels of HBV RNAs and antigens in human and mouse cells. HBV 1.2 (1 μg), siRNAs (20 nM), and/or IL-32γ (1 μg) plasmids were co-transfected into Huh7 cells or PMHs with Lipofectamine 2000. **a, b** At 72 h post-co-transfection of Huh7 cells (**a**) or PMH (**b**), the levels of HBV RNAs were determined by real-time PCR and the levels of HBeAg and HBsAg were determined by ELISA. Data were obtained from three independent experiments (mean ± S.D.). $p < 0.001$ by Student's t-test.

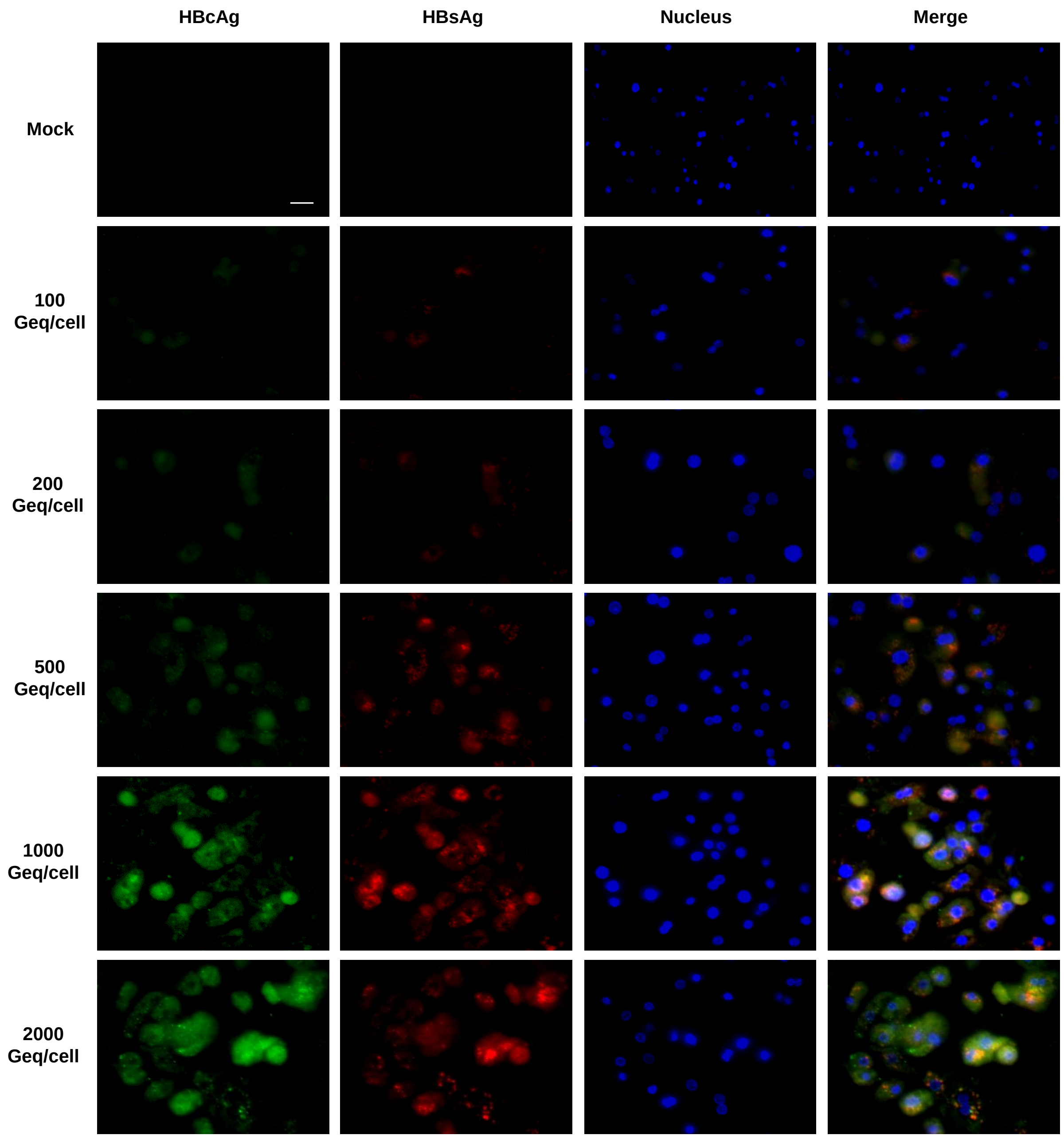

Supplementary Figure 10. Determination of optimal conditions for HBV infection in PHHs. PHHs were infected with HBV inoculum of 100, 200, 500, 1000, or 2000 HBV Geq/cell. At 9 days post-infection (dpi), expression levels of HBcAg and HBsAg were examined by confocal microscopy. Magnification, ×200 and scale bar, 50 μm.

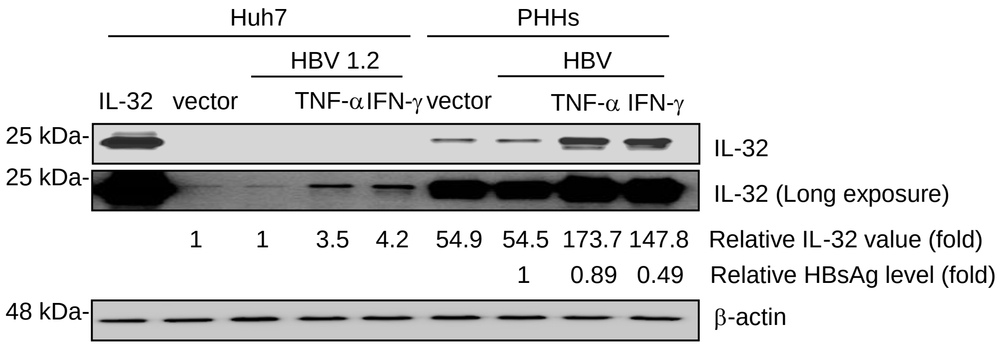

Supplementary Figure 11. Comparison of IL-32γ induction level by cytokines between Huh7 cells and PHHs. PHHs were infected with HBV and Huh7 cells were transfected with the HBV 1.2 plasmid. Both cell types were treated with TNF-α (20 ng/mL) or IFN-γ (1000 U/mL) for 2 days. Expression level of IL-32γ was determined by Western blotting. A lysate of cells ectopically expressing IL-32γ was used as a positive control.

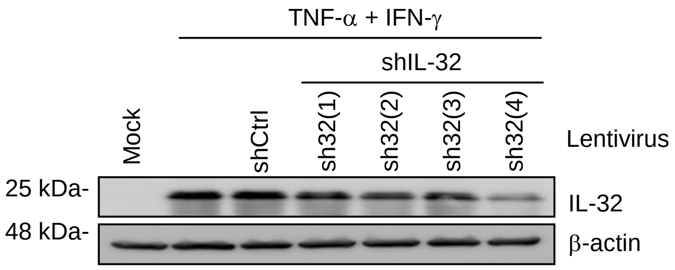

Supplementary Figure 12. Knock-down of cytokine-induced IL-32γ by shIL-32 lentiviruses. Huh7 cells were infected with shIL-32 lentiviruses. At 48 h post-infection, the level of cytokine-induced IL-32 protein was determined by Western blotting.

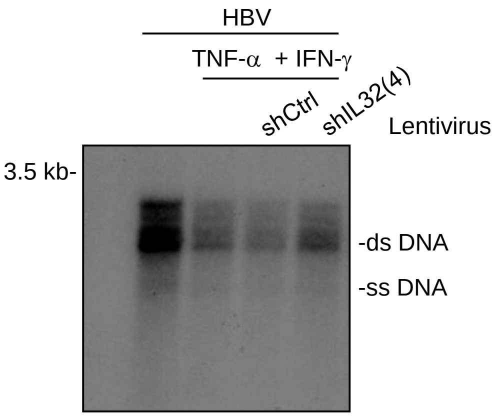

Supplementary Figure 13. Effect of IL-32γ knock-down on cytokine-mediated suppression of HBV in HepG2 cells. HBV 1.2 plasmid was transfected into HepG2 cells. Next day, the cells were infected with the control and shIL-32 lentiviruses. At 12 h post-infection, TNF-α (20 ng/mL) and IFN-γ (500 U/mL) were added for 2 days. HBV DNA was analyzed by Southern blotting.

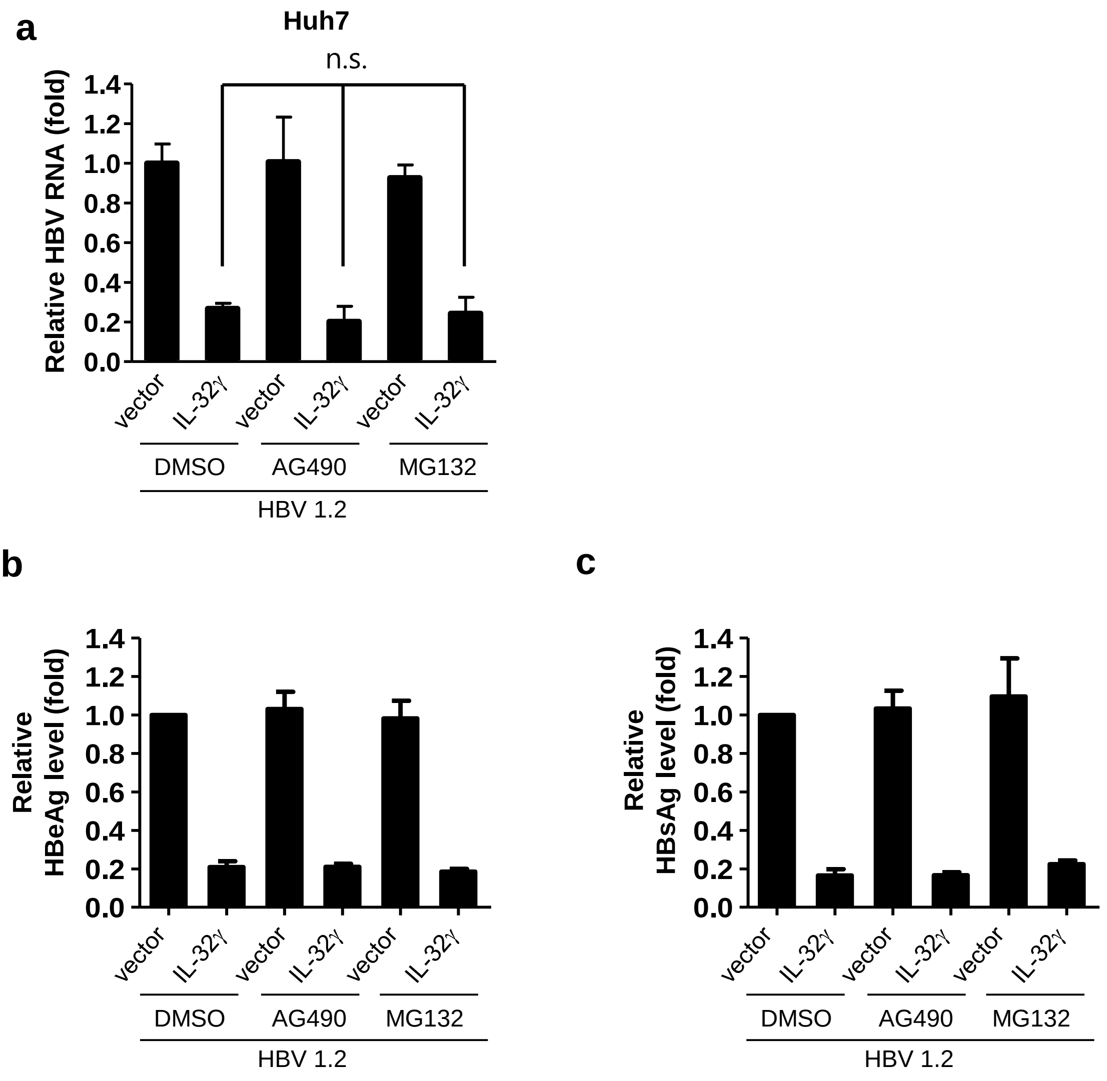

Supplementary Figure 14. Effect of kinase and proteasome inhibitors on IL-32-mediated suppression of HBV.

HBV 1.2 (1 μg) and the IL-32 plasmid (1 μg) were co-transfected into Huh7 cells. At 3 days post-transfection, the cells were treated with the inhibitors of JAK (20 μM AG490) and proteasome (20 μM MG132) for 12 h, and the cells and supernatants were collected. **a** The level of HBV mRNA was analyzed by real-time PCR. **b, c** The levels of HBeAg and HBsAg were determined by ELISA. Data were obtained from three independent experiments (mean ± S.D.). n.s, no significant.

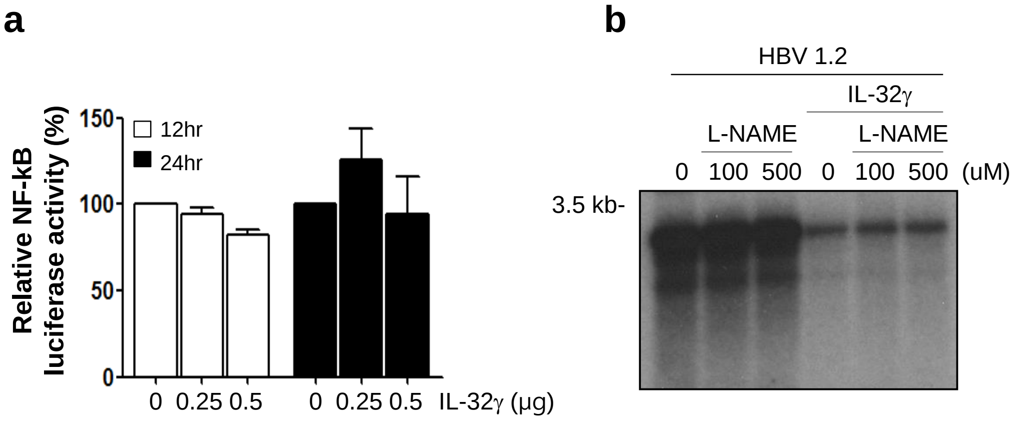

Supplementary Figure 15. NF-κB activity and nitric oxide production are not involved in IL-32γ-mediated inhibition of HBV. (a) Huh7 cells were co-transfected with NF-κB reporter and IL-32γ plasmid, and NF-κB activity was determined from luciferase activity. (b) Effect of nitric oxide synthase (NOS) inhibitor on IL-32γ-mediated inhibition of HBV. Data were obtained from three independent experiments (mean ± S.D.).

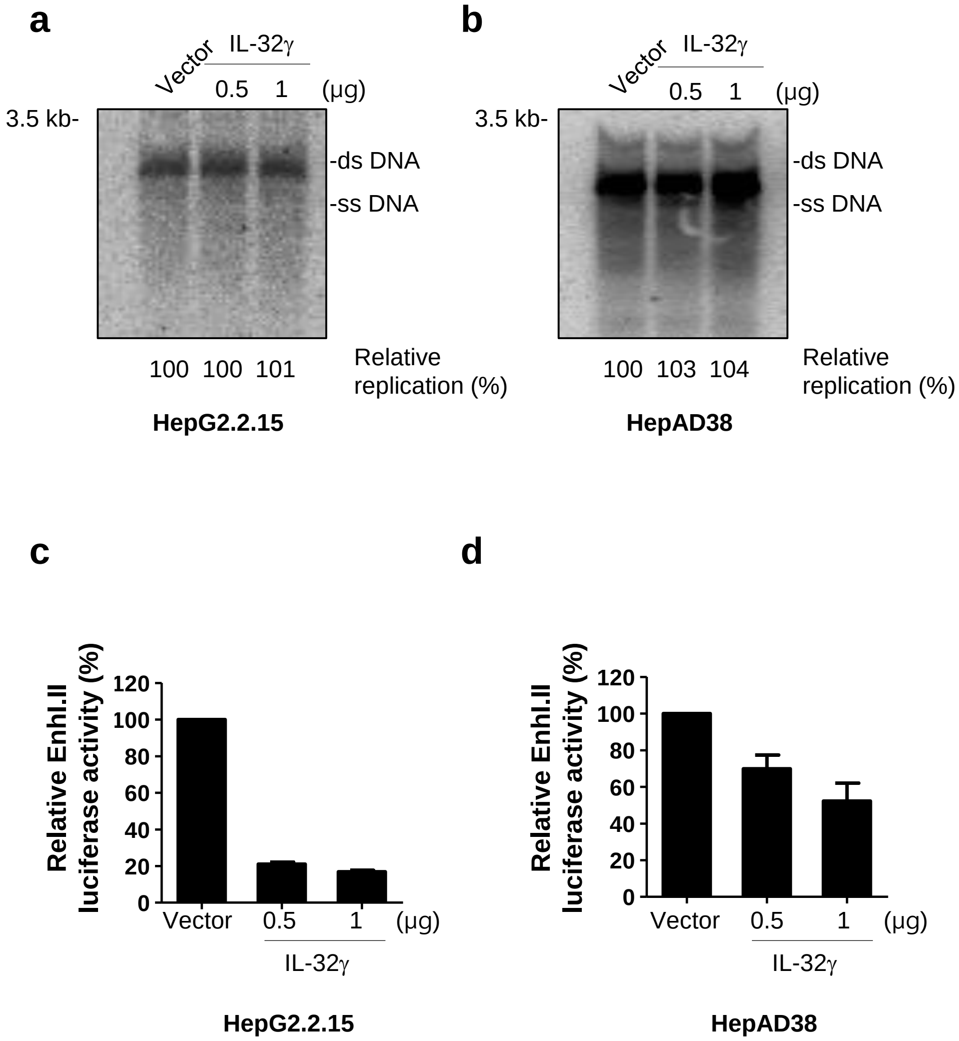

Supplementary Figure 16. Effect of IL-32 in HBV stable cell lines.

a, b HepG2.2.15 or HepAD38 stable cells were transfected with IL-32γ or empty plasmid. At 72 h post-transfection, the level of HBV replication was determined by Southern blotting. **c, d** Effect of IL-32γ on the enhancer reporter in HepG2.2.15 and HepAD38 cells. Cells were co-transfected with IL-32γ and reporter plasmids, and relative luciferase activity was determined at 48 h post-co-transfection using a Steady Glo-Luciferase assay system. Data were obtained from three independent experiments (mean ± S.D.).

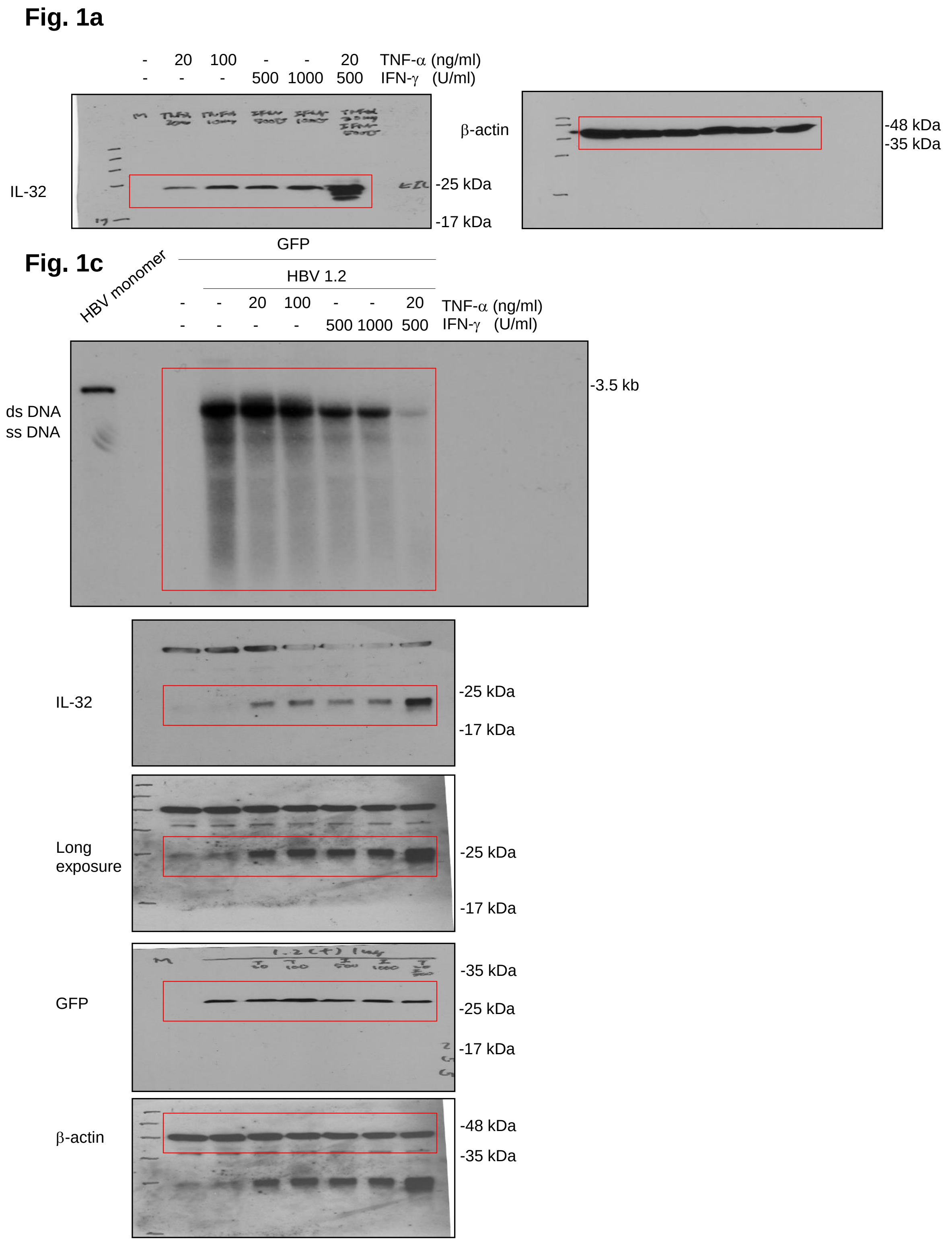

Supplementary Figure 17. Original uncropped data. Original uncropped Western blot/Southern blot of Fig. 1a and Fig. 1c. Rectangles indicate cropped parts used in respective figures.

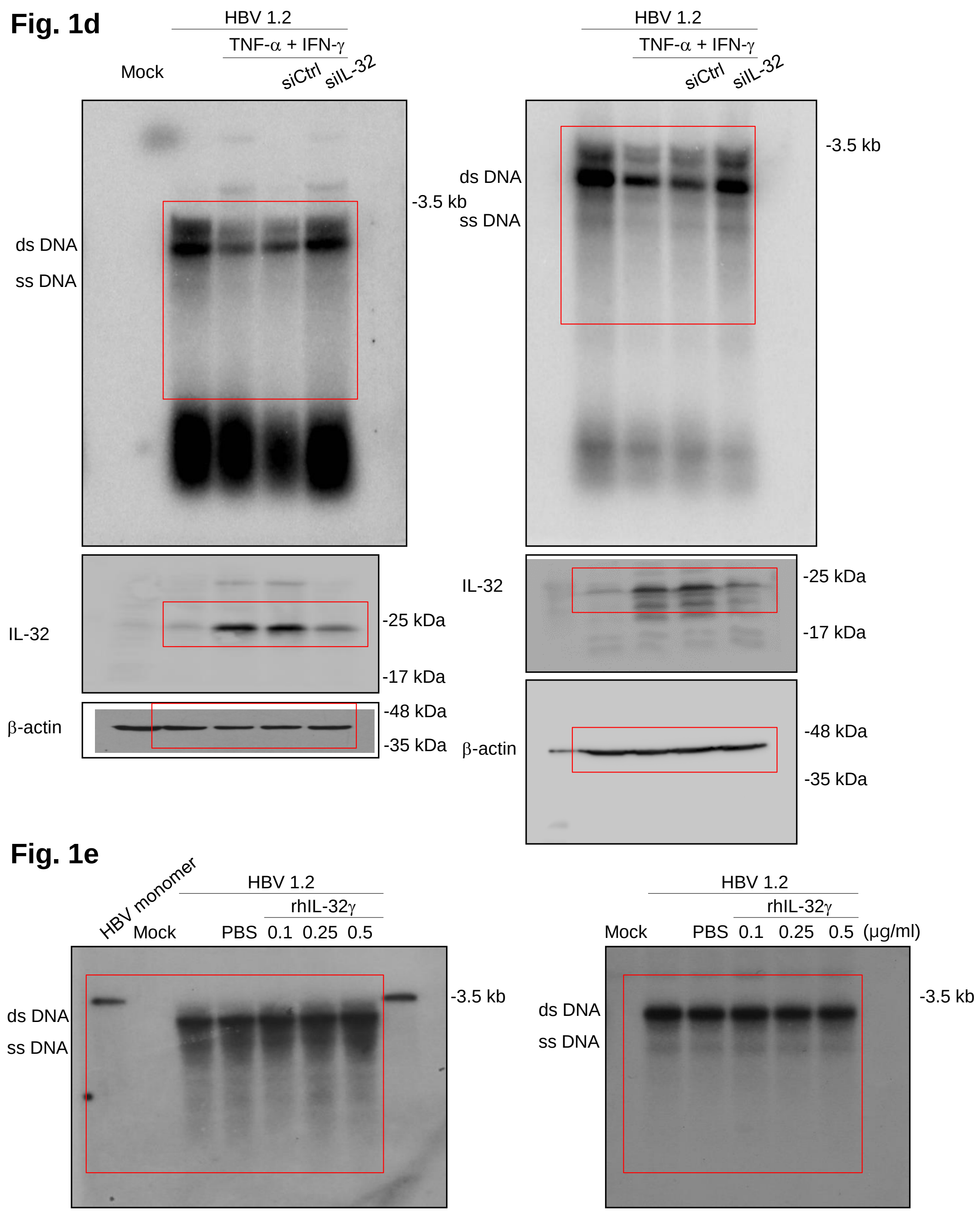

Supplementary Figure 18. Original uncropped data. Original uncropped Western blot/Southern blot of Fig. 1d and Fig. 1e. Rectangles indicate cropped parts used in respective figures.

Fig. 2a

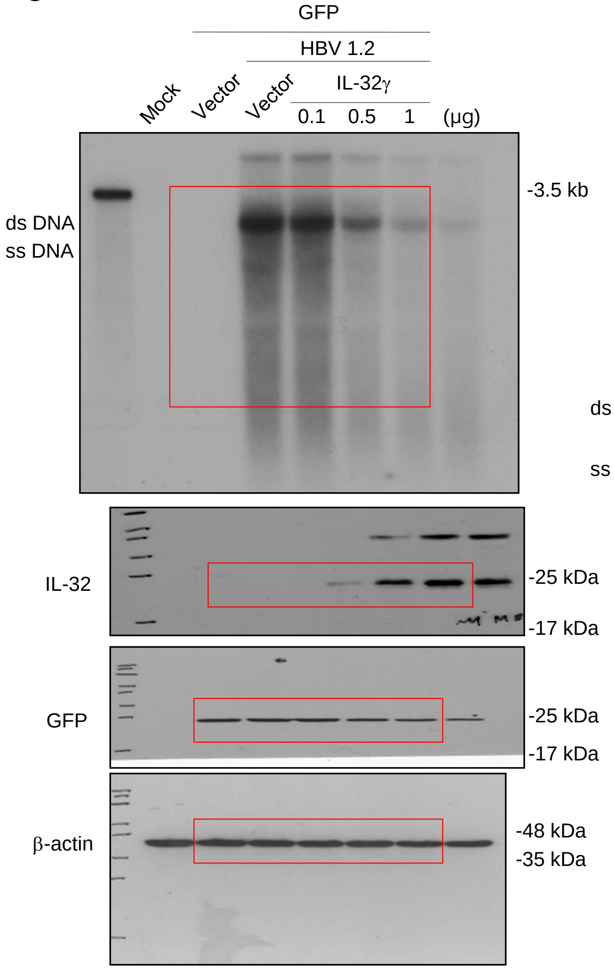

Fig. 2d

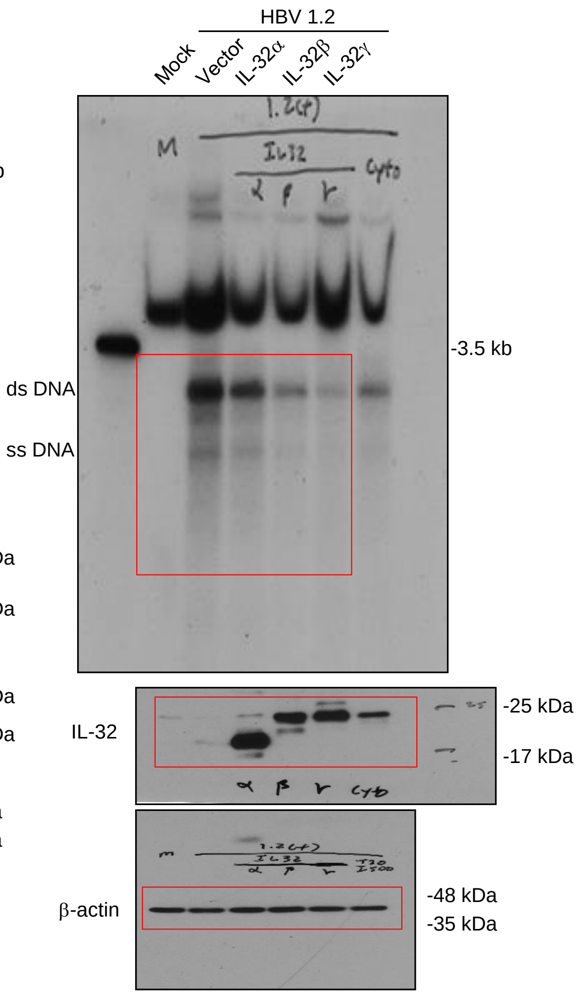

Supplementary Figure 19. Original uncropped data. Original uncropped Western blot/Southern blot of Fig. 2a and Fig. 2d. Rectangles indicate cropped parts used in respective figures.

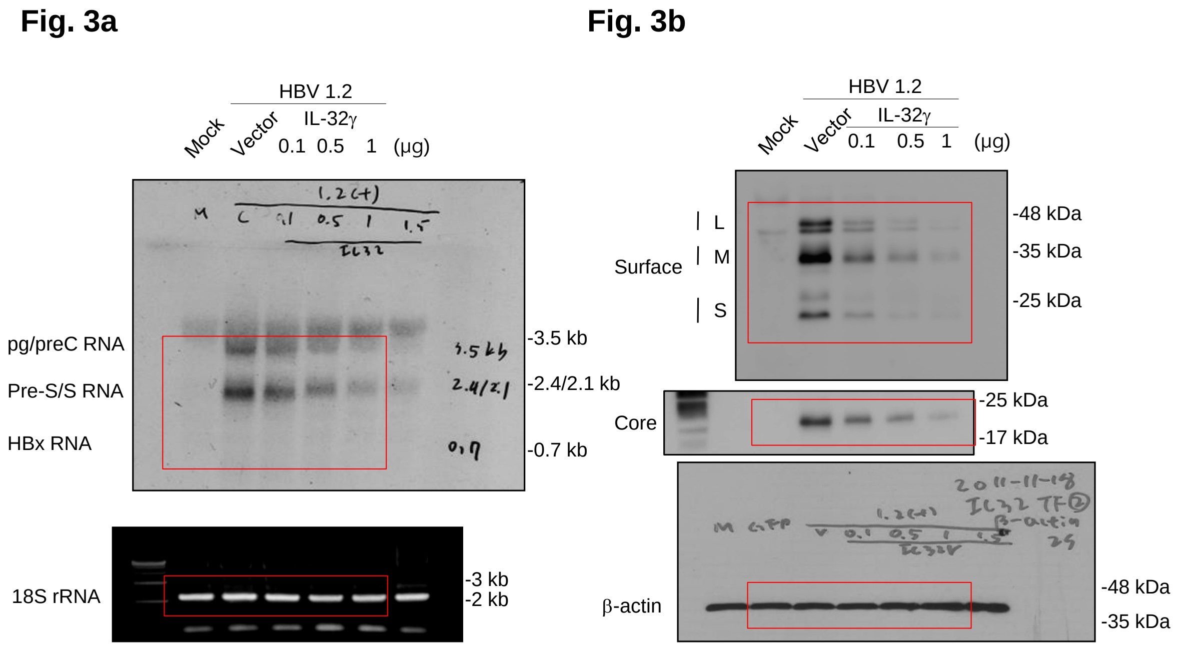

Supplementary Figure 20. Original uncropped data. Original uncropped Western blot/Norhern blot of Fig. 3a and Fig. 3b. Rectangles indicate cropped parts used in respective figures.

Fig. 3e

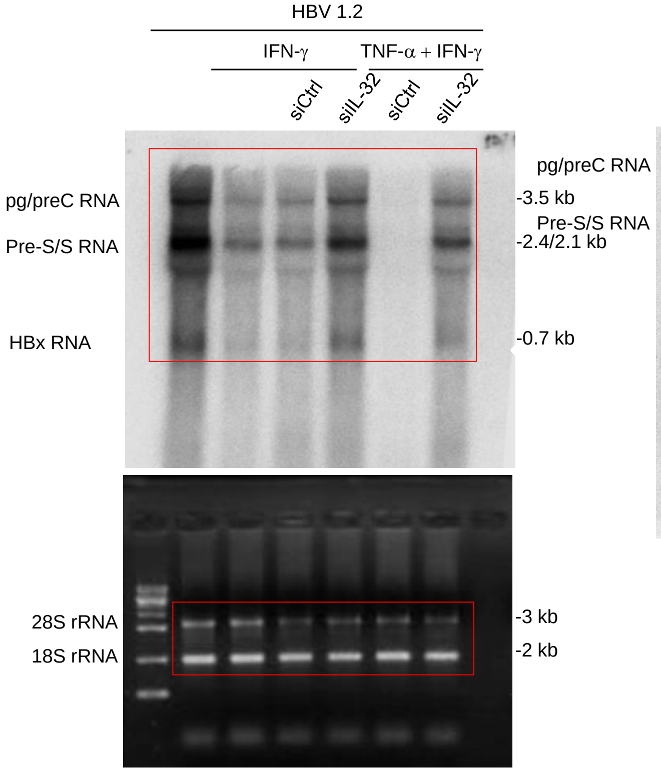

Fig. 3f

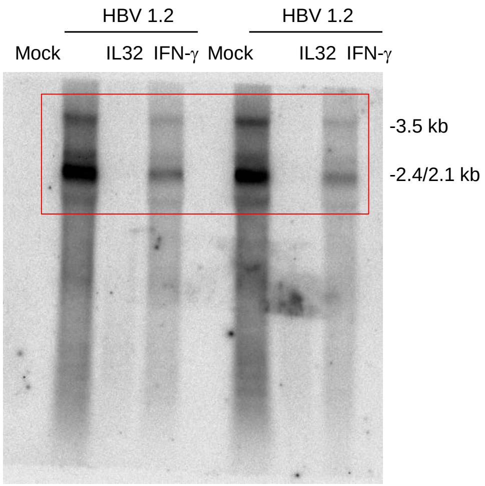

Fig. 3g

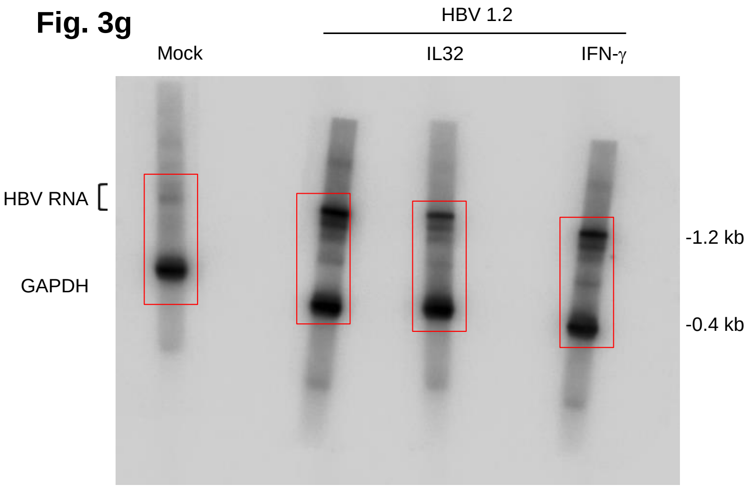

Supplementary Figure 21. Original uncropped data. Original uncropped Northern blot/Run-on assay of Fig. 3e, Fig. 3f and Fig. 3g. Rectangles indicate cropped parts used in respective figures.

Fig. 4b

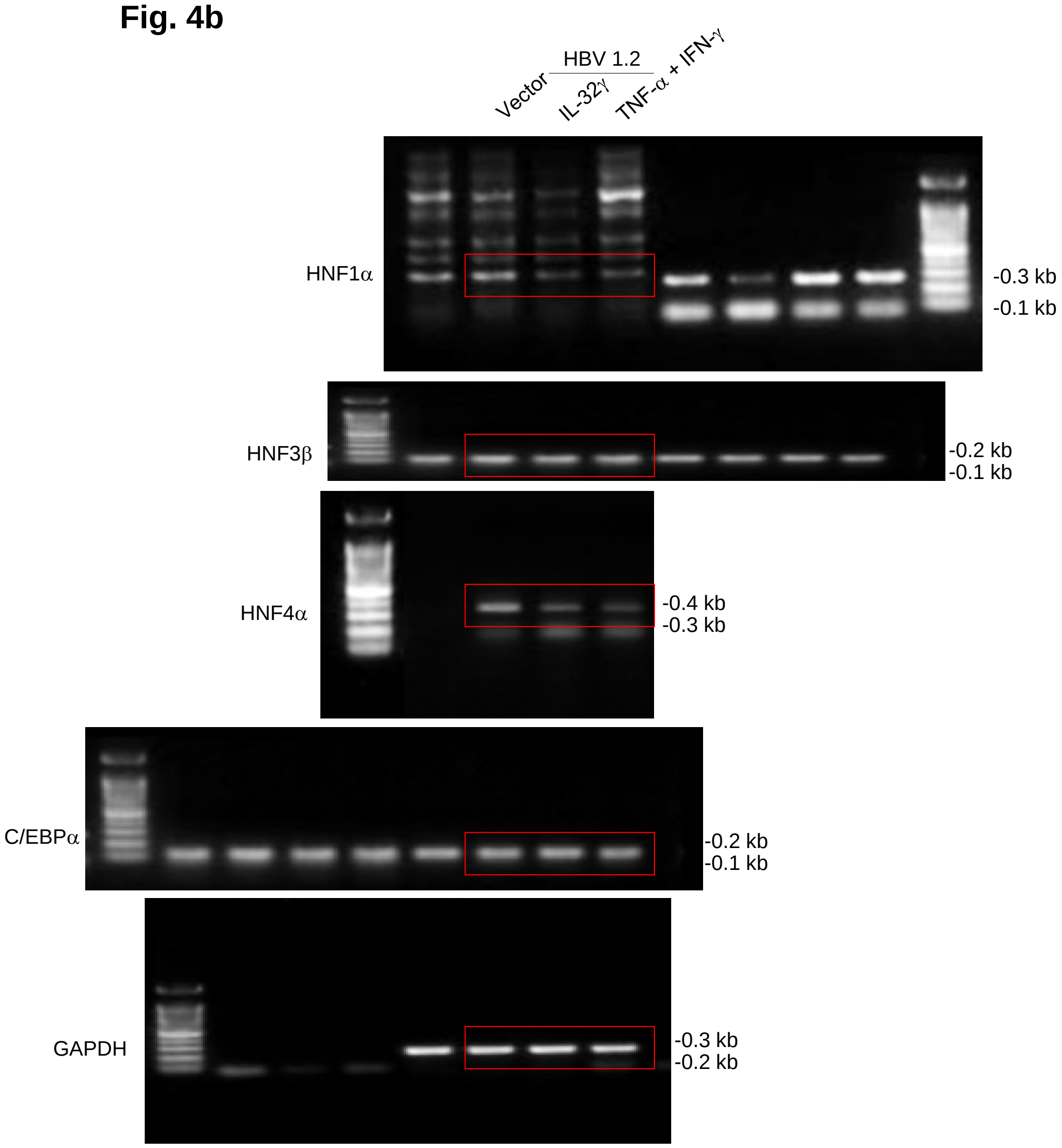

Supplementary Figure 22. Original uncropped data. Original uncropped PCR of Fig. 4b. Rectangles indicate cropped parts used in respective figures.

Fig. 4d

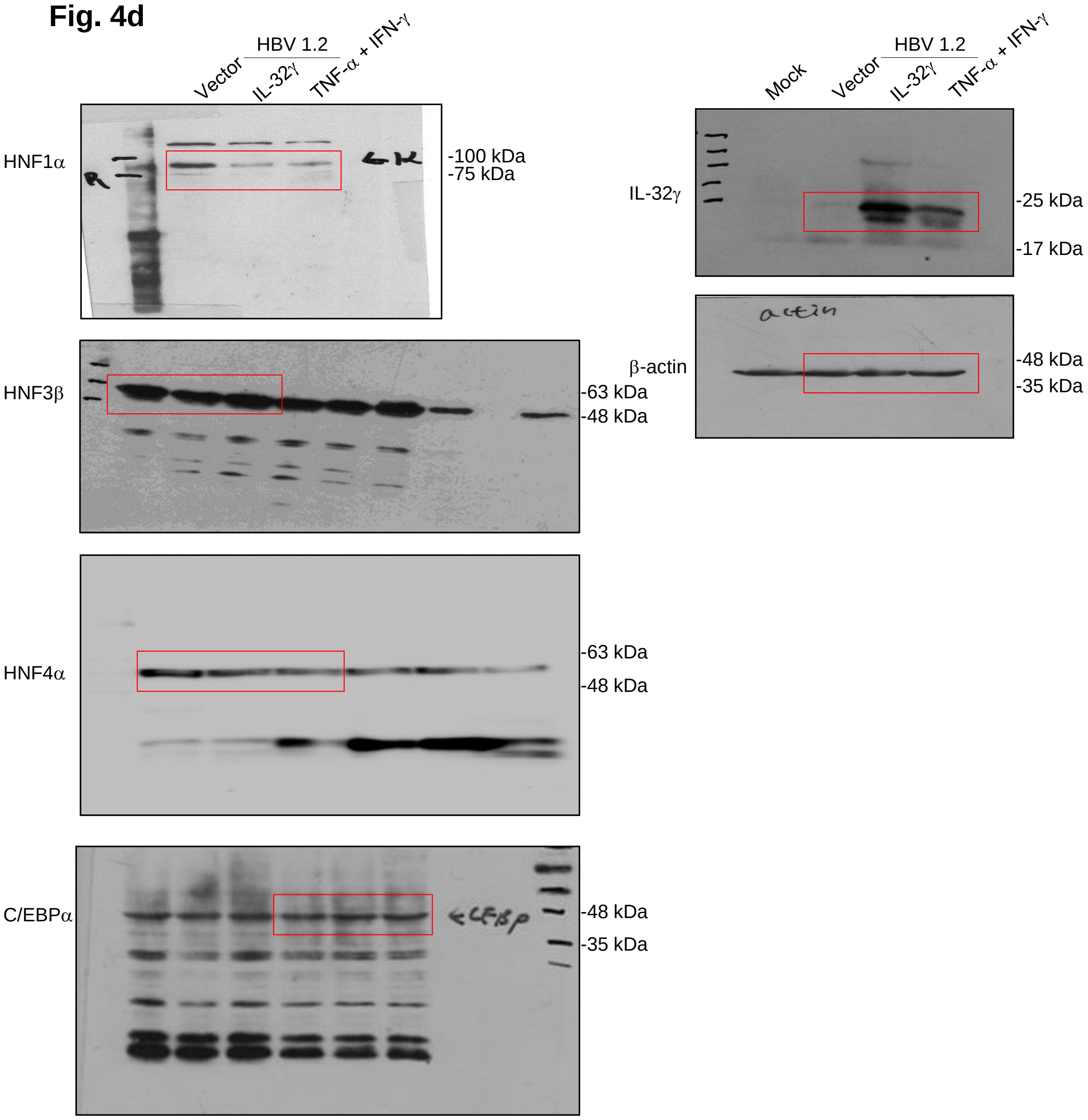

Supplementary Figure 23. Original uncropped data. Original uncropped Western blot of Fig. 4d. Rectangles indicate cropped parts used in respective figures.

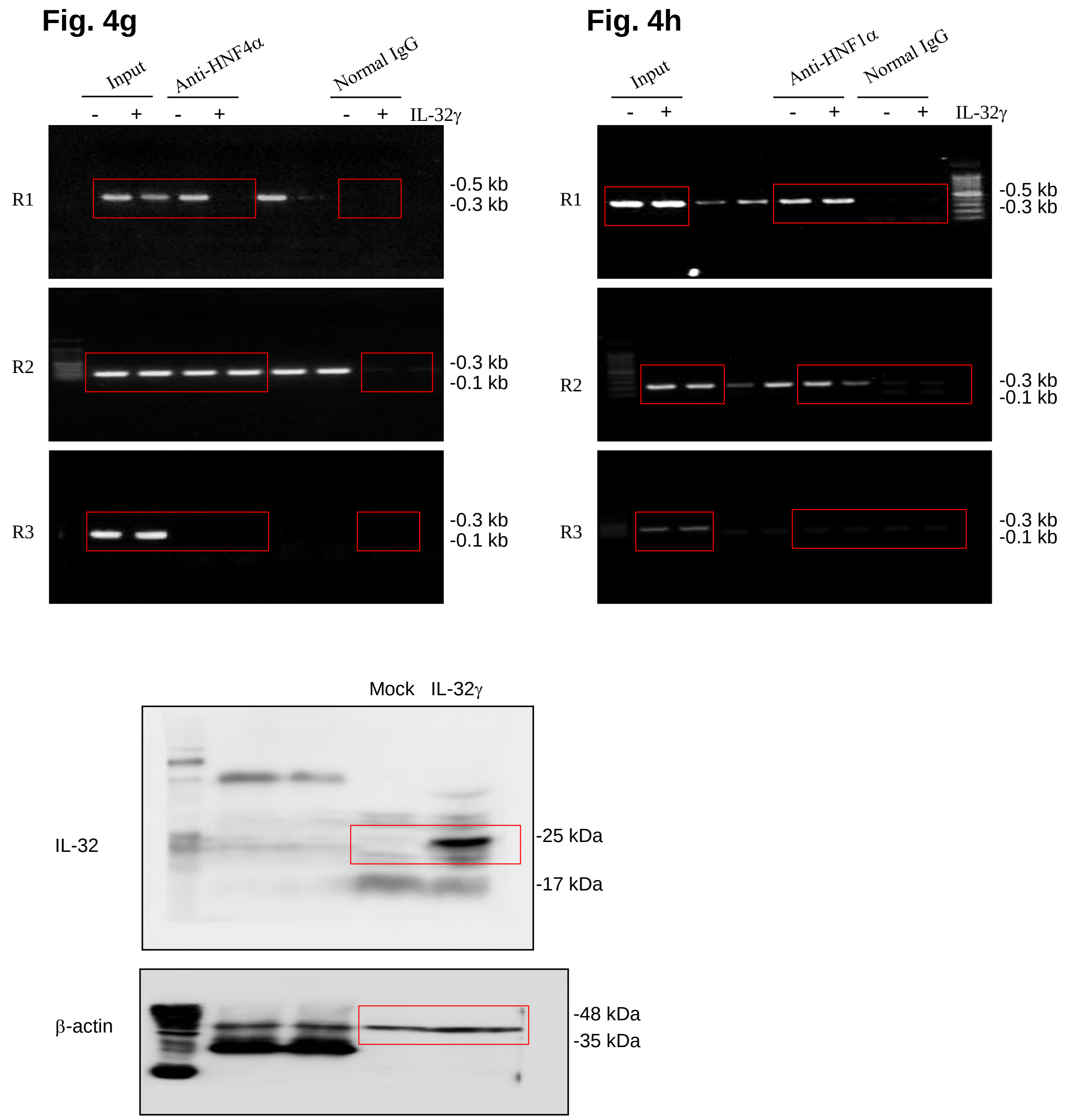

Supplementary Figure 24. Original uncropped data. Original uncropped Western blot/PCR of Fig. 4g, and Fig. 4h. Rectangles indicate cropped parts used in respective figures.

Fig. 4i

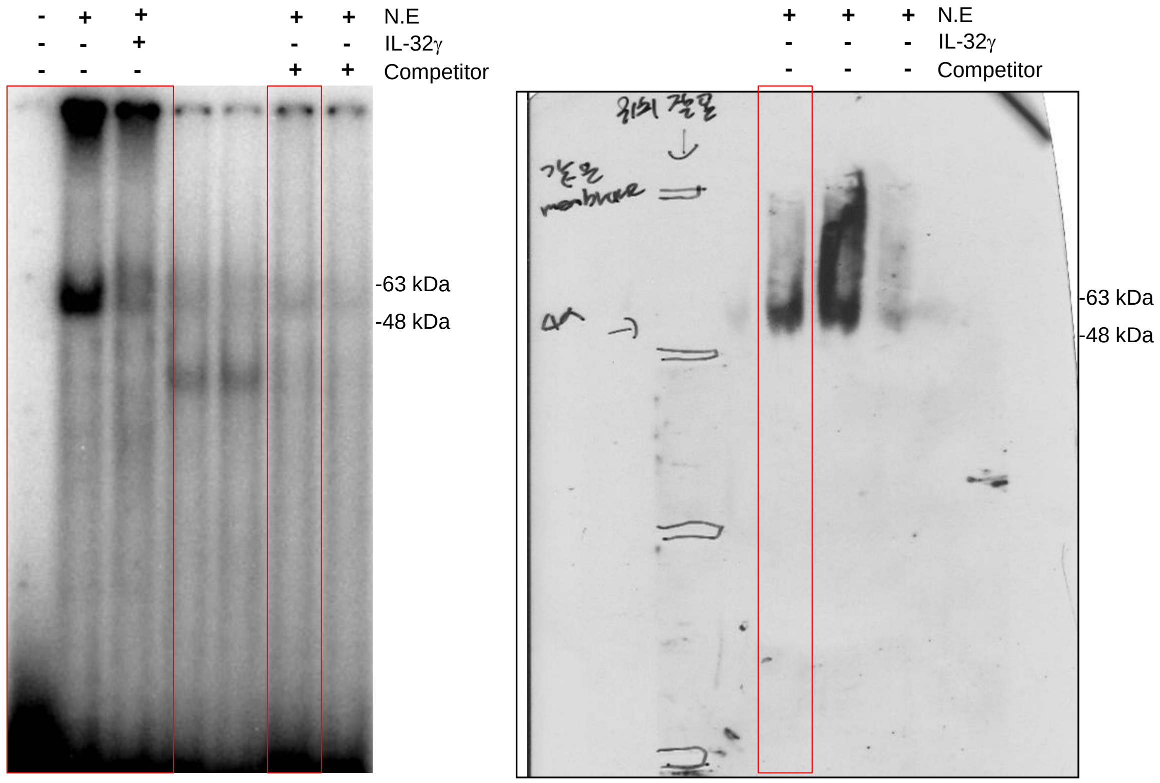

Supplementary Figure 25. Original uncropped data. Original uncropped native gel Western blot/EMSA of Fig. 4i. Rectangles indicate cropped parts used in respective figures.

Fig. 5a

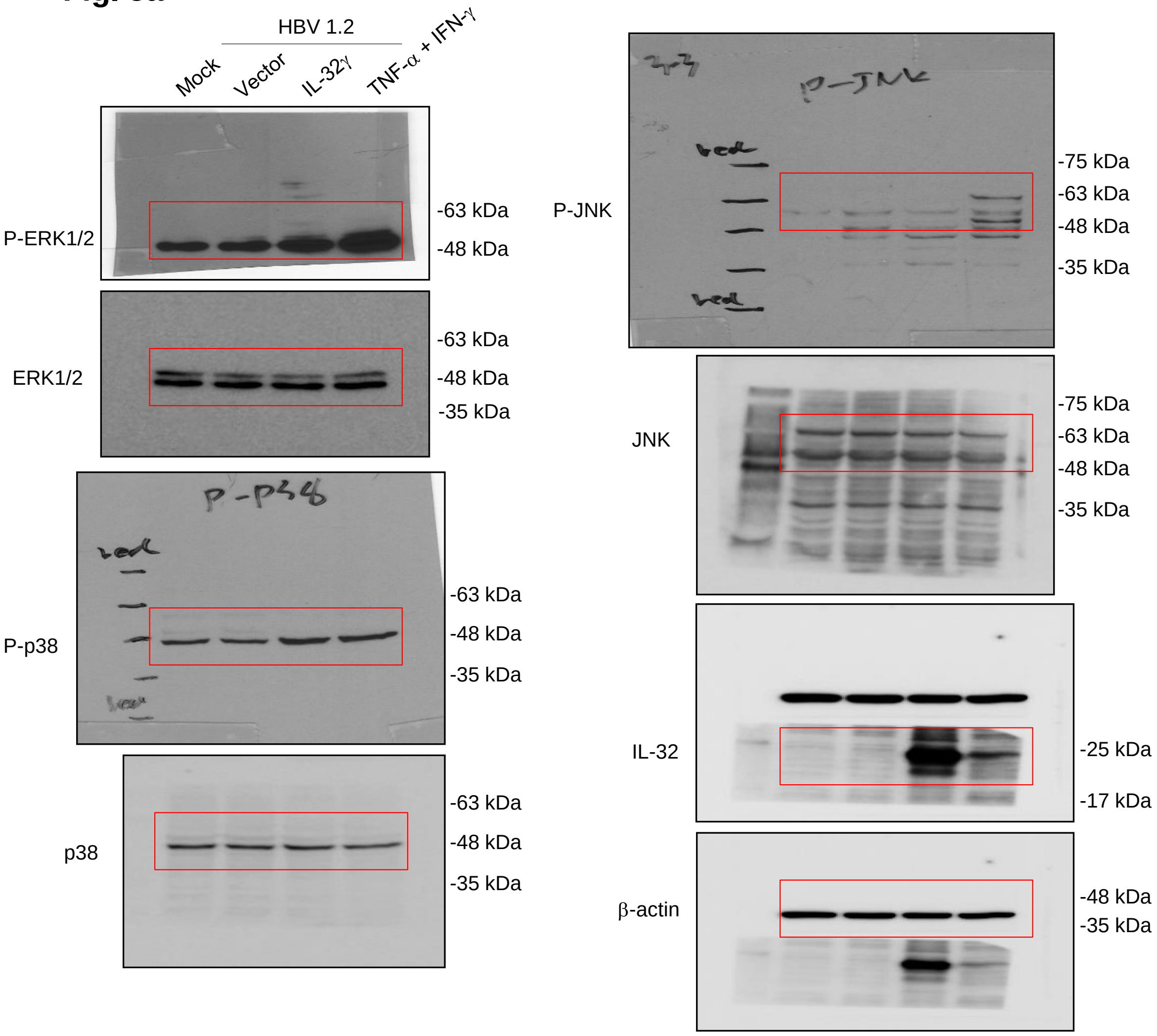

Supplementary Figure 26. Original uncropped data. Original uncropped Western blot of Fig. 5a. Rectangles indicate cropped parts used in respective figures.

Fig. 5b

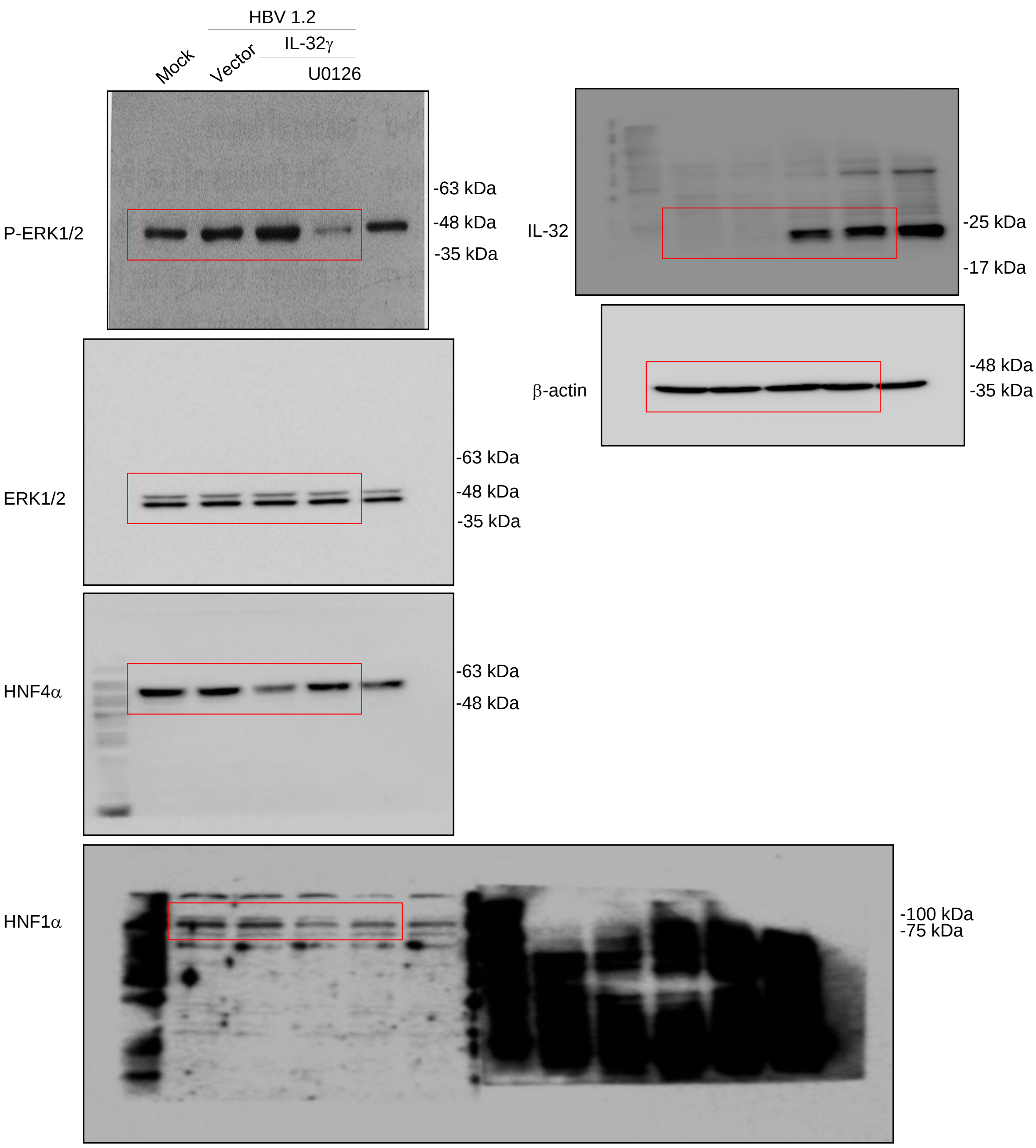

Supplementary Figure 27. Original uncropped data. Original uncropped Western blot of Fig. 5b. Rectangles indicate cropped parts used in respective figures.

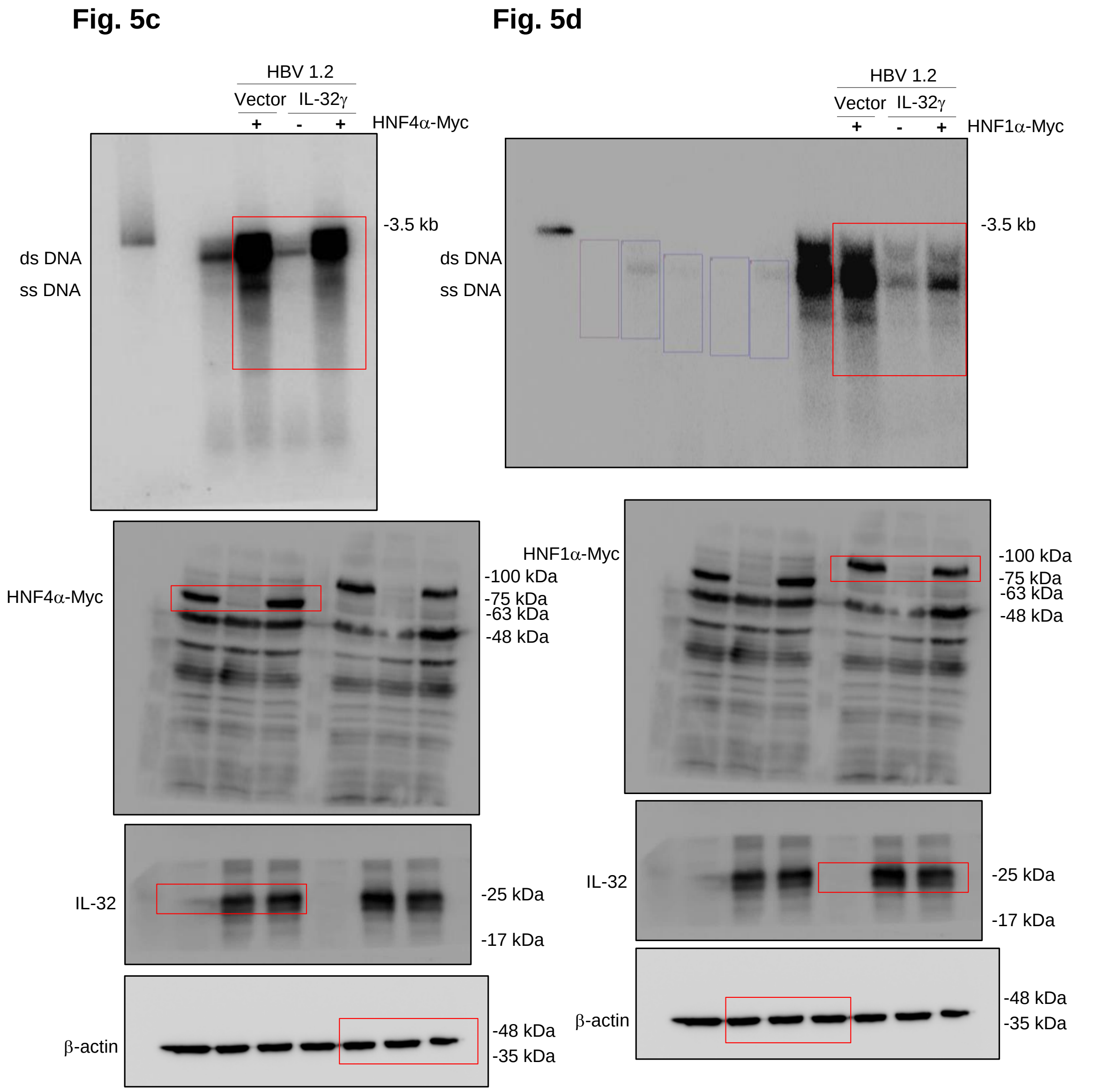

Supplementary Figure 28. Original uncropped data. Original uncropped Western blot/Southern blot of Fig. 5c and Fig. 5d. Rectangles indicate cropped parts used in respective figures.

Fig. 5e

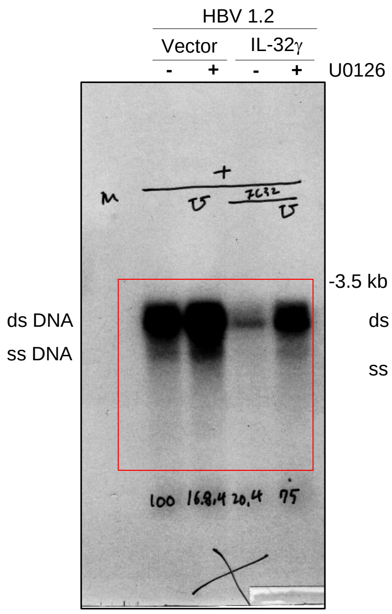

Fig. 6a

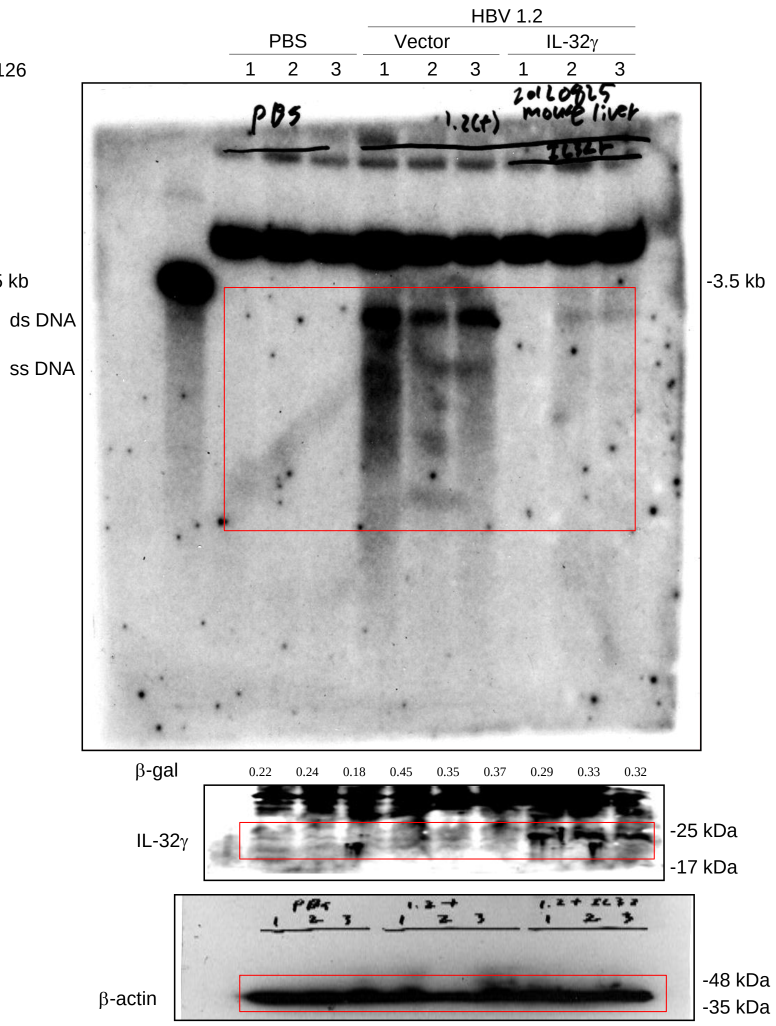

Supplementary Figure 29. Original uncropped data. Original uncropped Western blot/Southern blot of Fig. 5e and Fig. 6a. Rectangles indicate cropped parts used in respective figures.

Fig. 7b

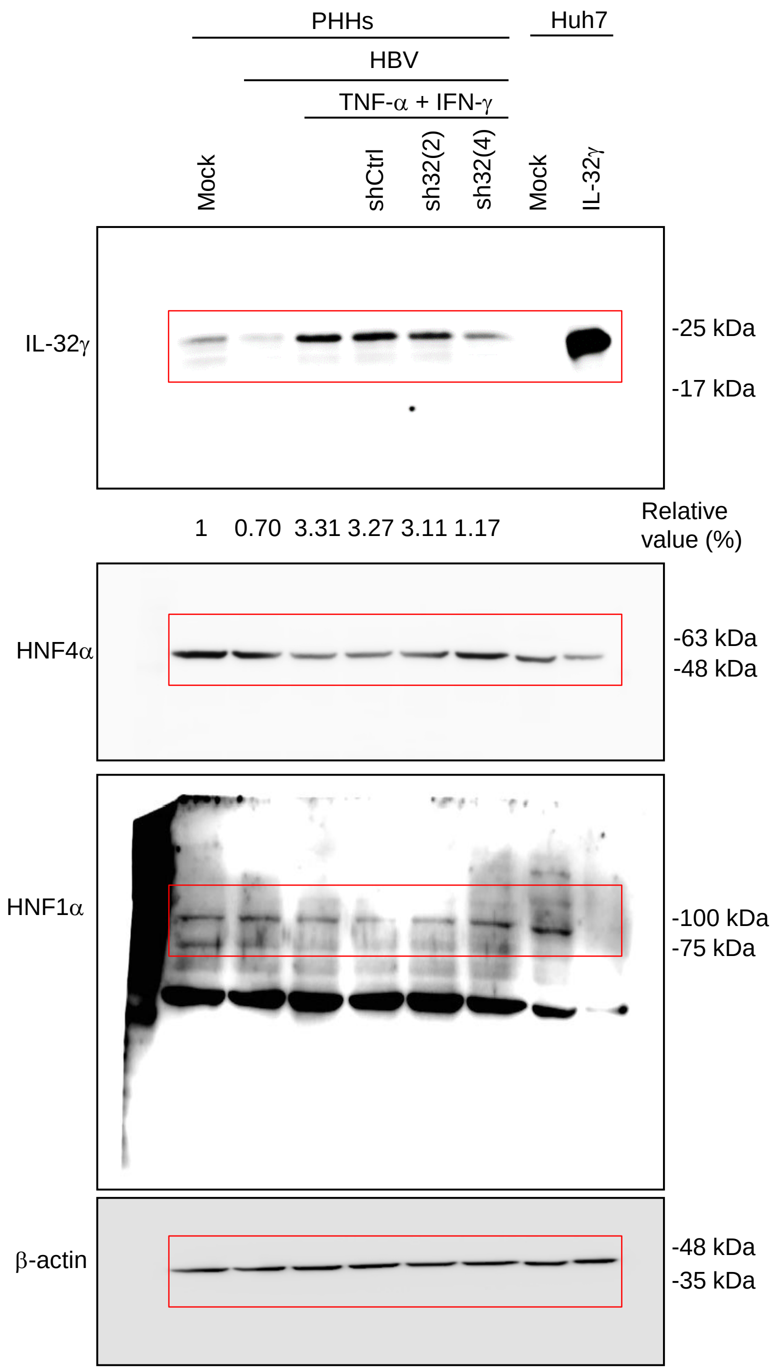

Supplementary Figure 30. Original uncropped data. Original uncropped Western blot of Fig. 7b. Rectangles indicate cropped parts used in respective figures.

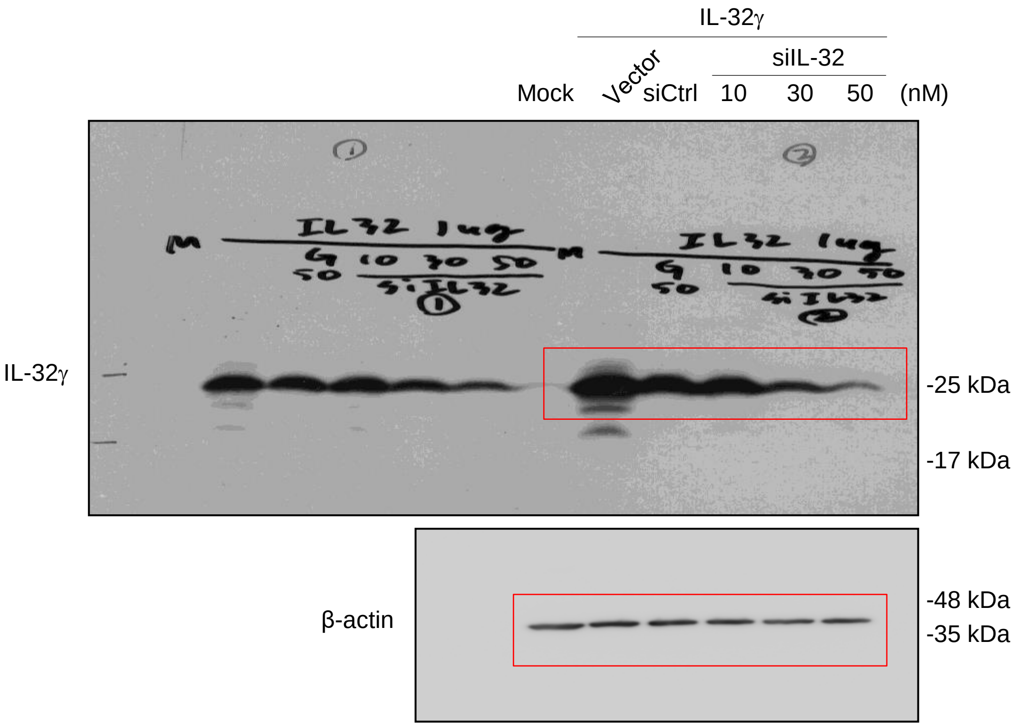

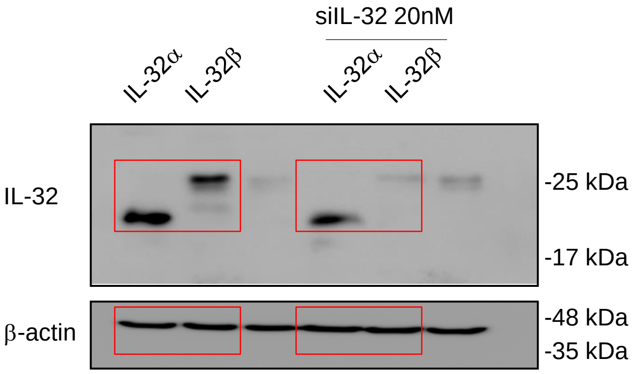

Supplementary Figure 31. Original uncropped data. Original uncropped Western blot of Supplementary Fig. 1. Rectangles indicate cropped parts used in respective figures.

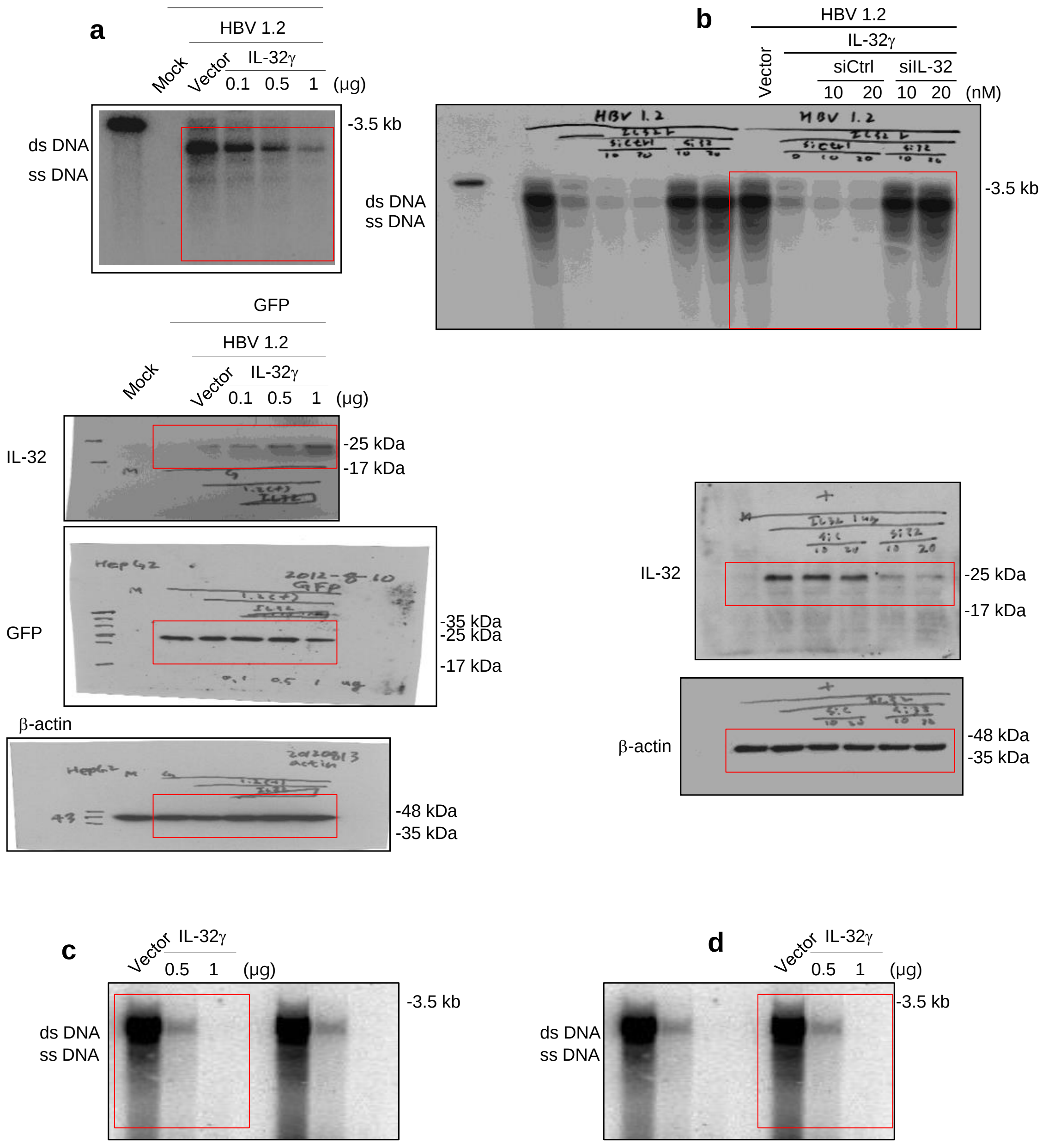

Supplementary Figure 32. Original uncropped data. Original uncropped Western blot/Southern blot of Supplementary Fig. 3. Rectangles indicate cropped parts used in respective figures.

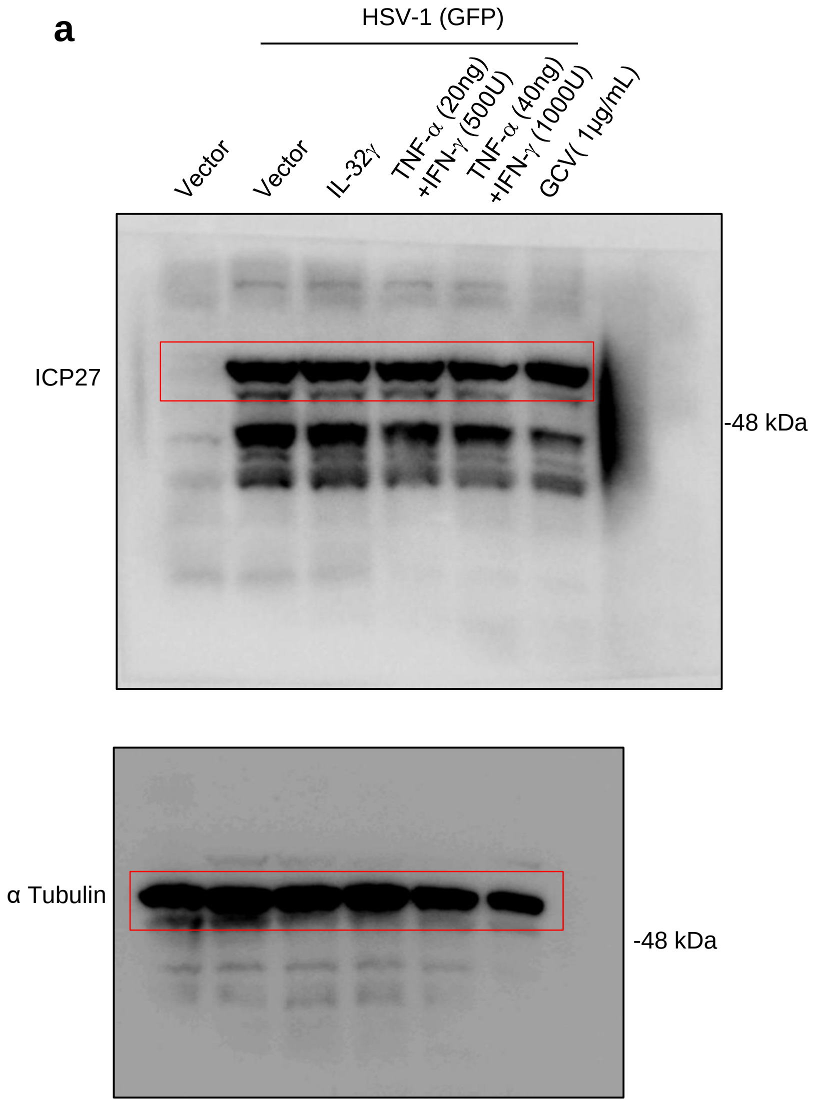

Supplementary Figure 33. Original uncropped data. Original uncropped Western blot of Supplementary Fig. 4. Rectangles indicate cropped parts used in respective figures.

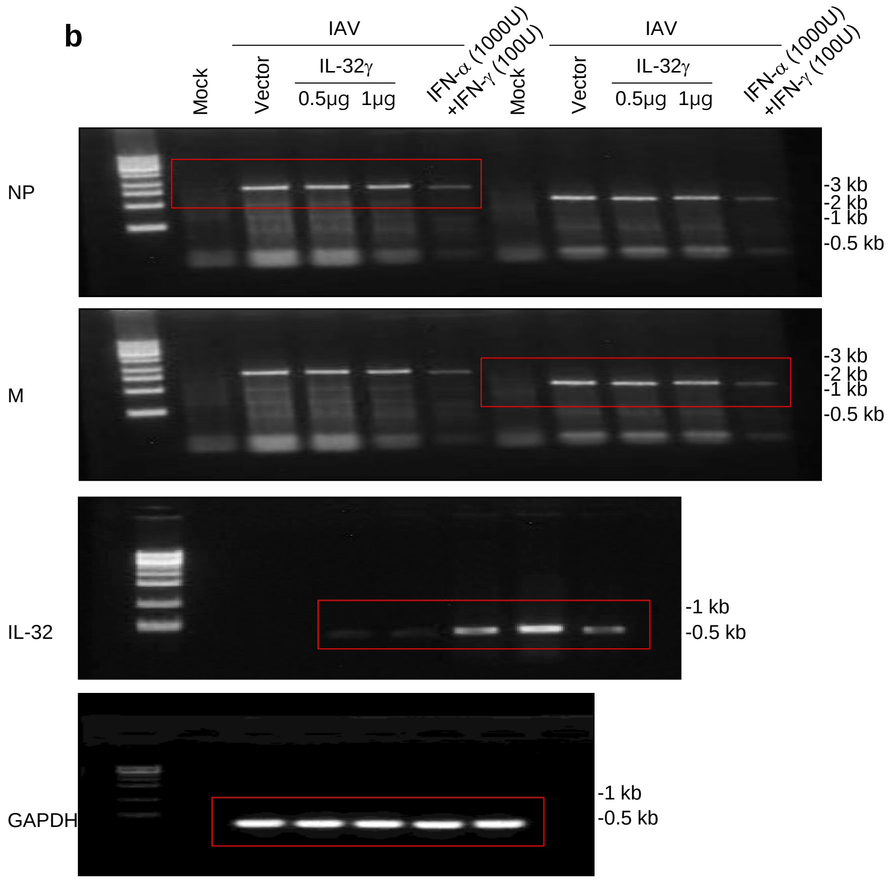

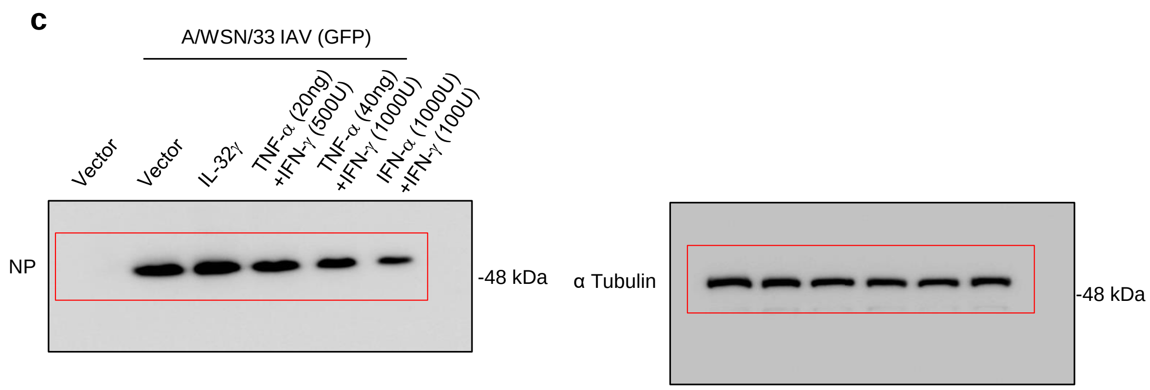

Supplementary Figure 34. Original uncropped data. Original uncropped Western blot/PCR of Supplementary Fig. 5. Rectangles indicate cropped parts used in respective figures.

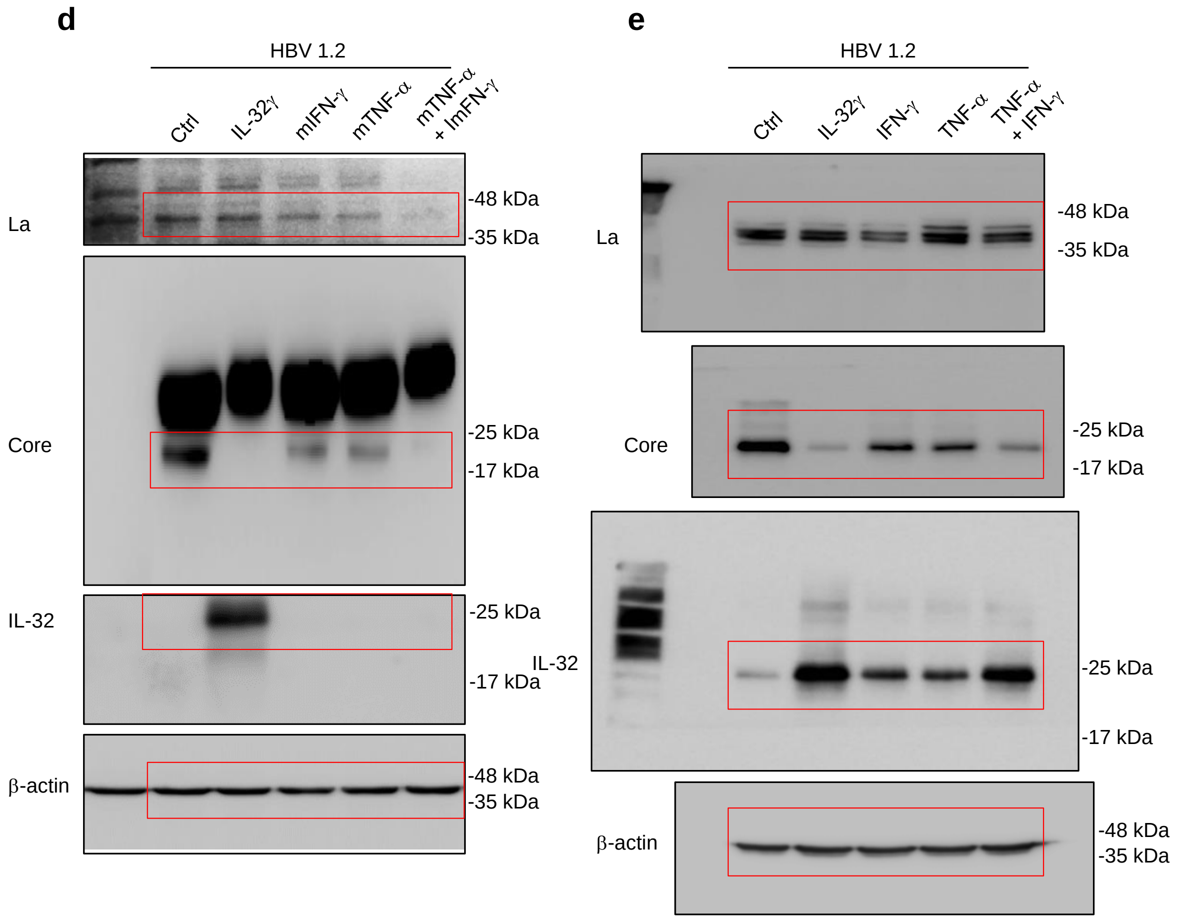

Supplementary Figure 35. Original uncropped data. Original uncropped Western blot of Supplementary Fig. 7. Rectangles indicate cropped parts used in respective figures.

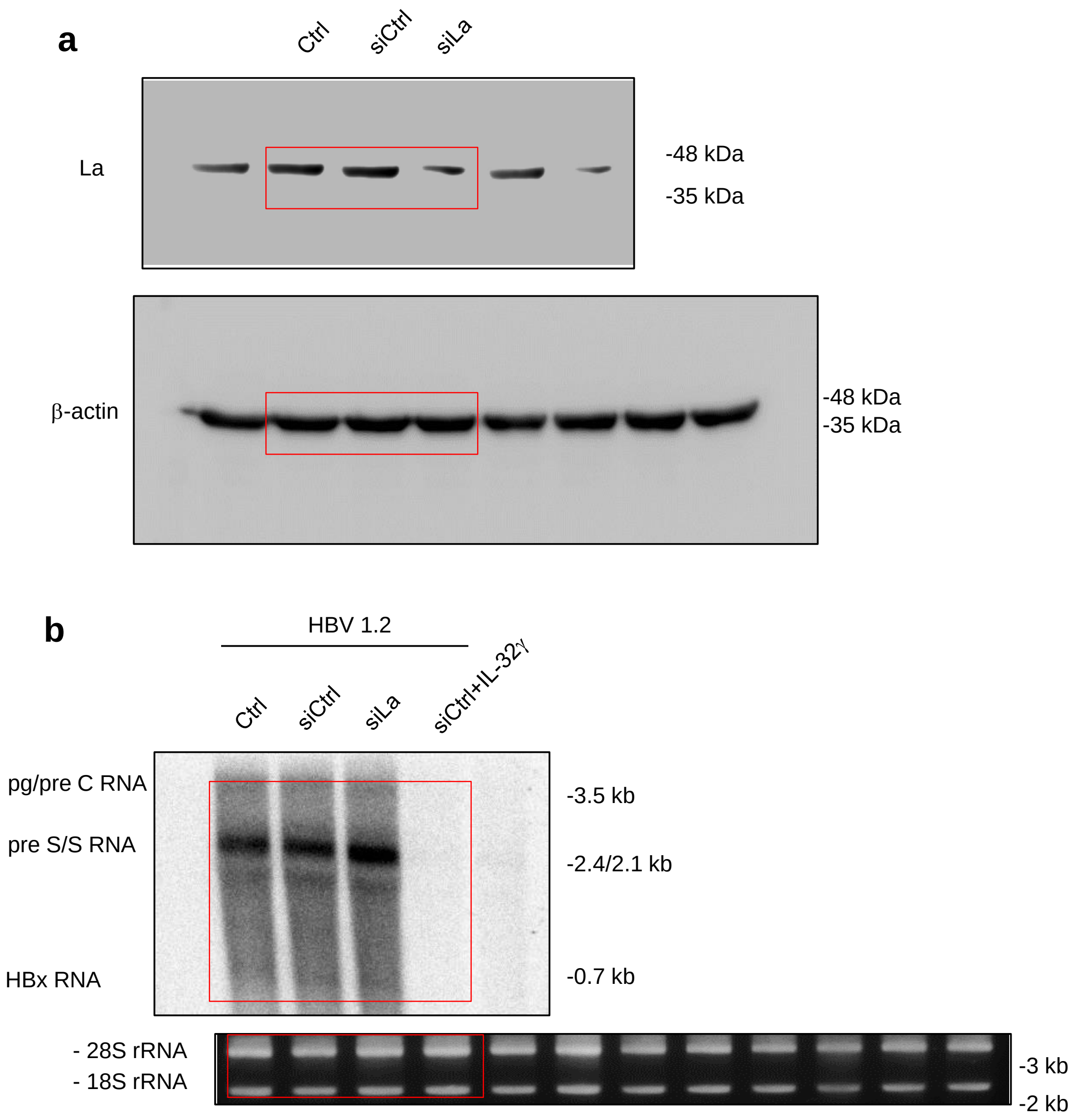

Supplementary Figure 36. Original uncropped data. Original uncropped Western blot/Northern blot of Supplementary Fig. 8. Rectangles indicate cropped parts used in respective figures.

Supplementary Fig. 11

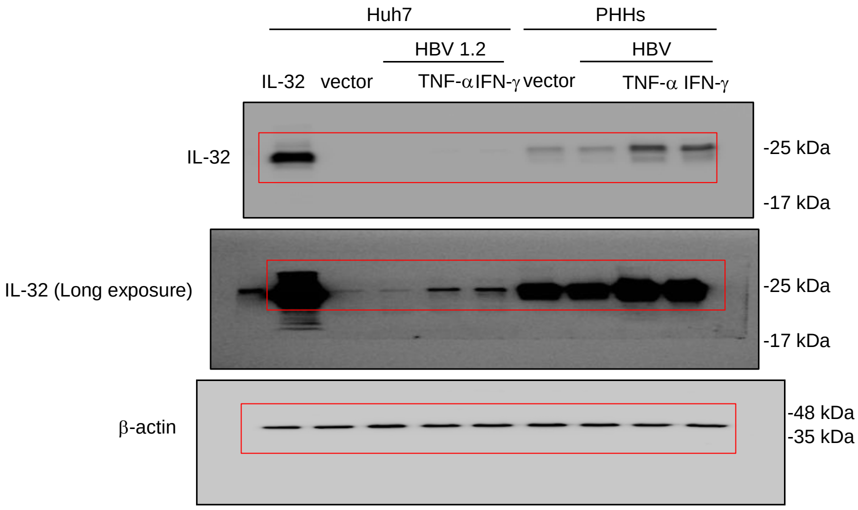

Supplementary Fig. 12

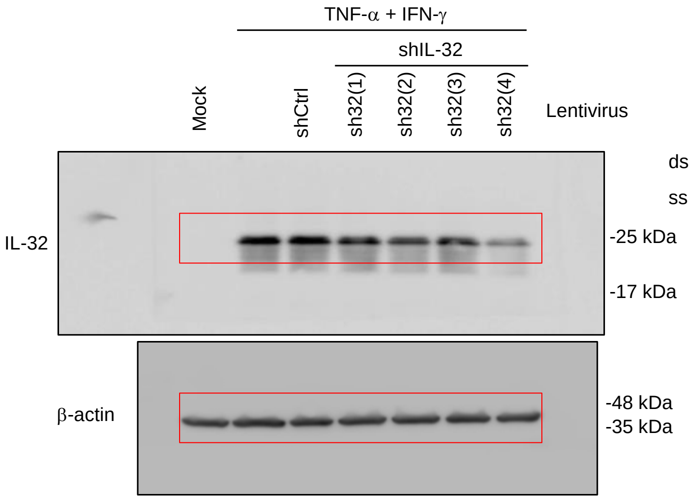

Supplementary Fig. 13

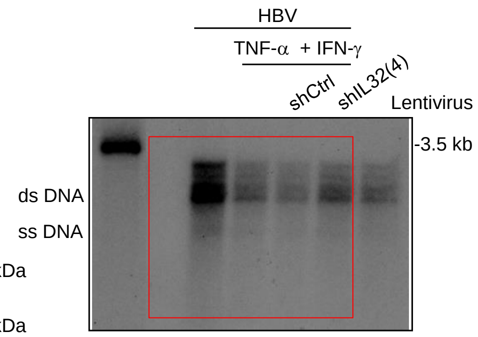

Supplementary Figure 37. Original uncropped data. Original uncropped Western blot/Southern blot of Supplementary Fig. 11, Fig. 12, and Fig. 13. Rectangles indicate cropped parts used in respective figures.

Supplementary Fig. 15

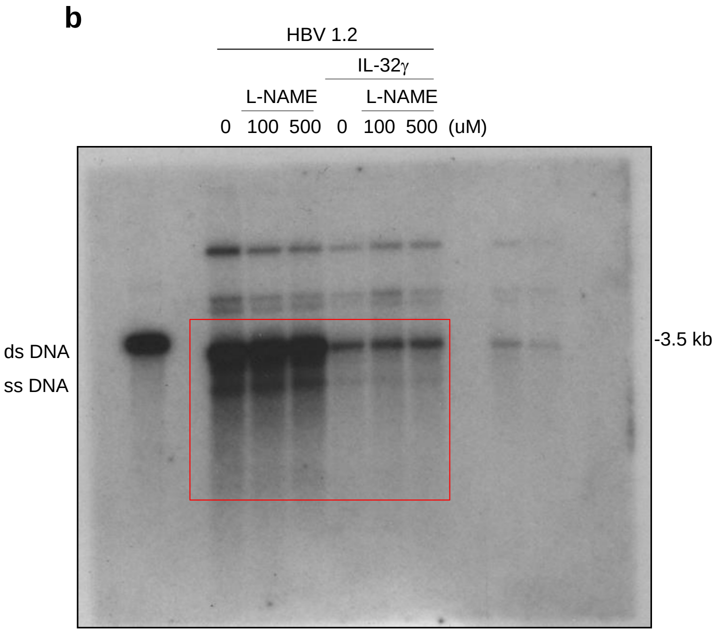

Supplementary Fig. 16

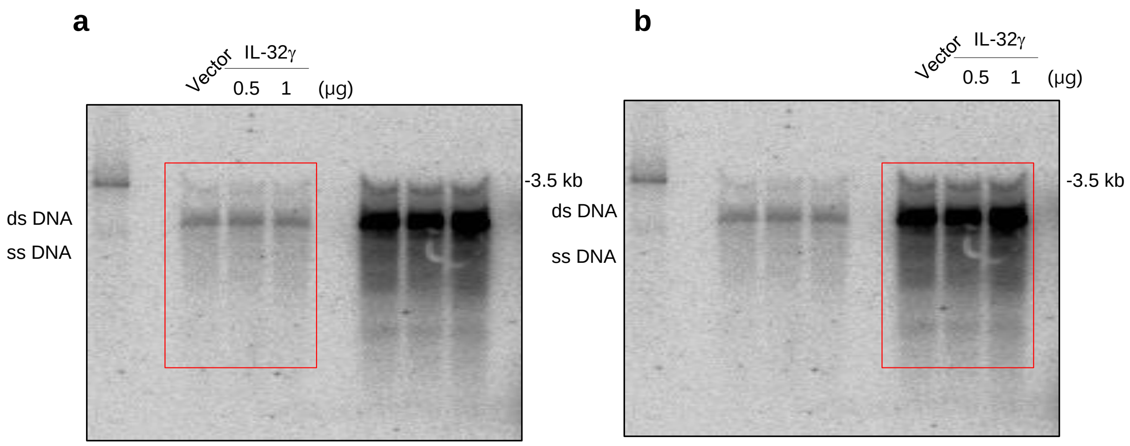

Supplementary Figure 38. Original uncropped data. Original uncropped Southern blot of Supplementary Fig. 15 and Fig. 16. Rectangles indicate cropped parts used in respective figures.

Supplementary Table. 1 Primer sequences used in this study

Gene name	Forward	Reverse
IL-32	5’-TGTGCGCTATGGACAGCCTGC-3’	5’-CTGTGTTGGTGAACGTAGGA-3’
HBV RC	5'-CTCGTGGTGGACTTCTCTC-3'	5'-CTGCAGGATGAAGAGGAA-3'
HBx	5'-AAAAAGTTGCATGGTGCTGGTGAAC-3'	5'-GCGCGGGACGTACTTTGT-3'
GAPDH	5’-ATCATCCCTGCCTCTACTGG-3’	5’-TGGGTGTCGCTGTTGAAGTC-3’
HNF1α	5’-TGTGCGCTATGGACAGCCTGC-3’	5’-CTGTGTTGGTGAACGTAGGA-3’
HNF4α	5’-GAGTGGGCCAAGTACATCCCAG-3’	5’-GCTTTGAGGTAGGCATACT-3’
HNF3β	5’-AAGATGGAAGGGCACGAGC-3’	5’-TGTACGTGTTCATGCCGTTCA-3’
C/EBPα	5’-CCTTGTGCAATGTGAATGTGC-3’	5’-CGGAGAGTCTCATTTTGGCAA-3’
PKR	5'-AATGATGGAAAGCGAACAAGGAGTA-3'	5'-CTTCCACACAGTCAAGGTCCTTAGT-3'
MX1	5'-GCTTGCTTTCACAGATGTTTCG-3'	5'-AAGGGATGTGGCTGGAGATG-3'
ISG15	5’-GCTGGGACCTGACGGTG-3’	5’ -TTAGCTCCGCCCGCCAG-3’
Stat1	5'-CCGTTTTCATGACCTCCTGT-3'	5'-TGAATATTCCCCGACTGAGC-3'
Stat2	5'-GAGGCCTCAACTCAGACCAG-3'	5'-GCGTCCATCATTCCAGAGAT-3´
RNase L	5'-TCATTCATCGTCTCTTCCATCCT-3'	5'-ACATTCCGAAGCGTCCTATAGC-3'
IRF1	5'- CAAATCCCGGGGCTCATCTGG-3'	5'- CTGGCTCCTTTTCCCCTGCTTTGT-3'
OAS1	5'-TCCACCTGCTTCACAGAACTACA-3'	5'-TGGGCTGTGTTGAAATGTGTTT-3'
OAS2	5'-TACCTGAAGCCCTACGAAGAATG-3'	5'-TCAGCTTATCCCCAGTTTTATCG-3'
OAs3	5'-CCCTGGTCTGAGACTCACGTTT-3'	5'-GACTTGTGGCTTGGGTTTGAC-3'
OASL	5'-CGTGAAACATCGGCCAACTAAG-3'	5'-GTACCCATTTCCCAGGCATAGA-3'
IL-6	5’-AGCGCCTTCGGTCCAGTTGC-3’	5’-TGCCAGTGCCTCTTTGCTGC-3’
IL-β	5'-TCAGGCAGGCCGCGTCAGTT-3'	5'-TTGCTGTGAGTCCCGGAGCG-3'
TGF-β	5'-TGCCGCCCTTCTTCCCCTC-3'	5'-GGAGCACAAGCTGCCCACTGA-3'
TNF-α	5'-CCCTCTGGCCCAGGCAGTCA-3'	5'-GTAGGAAGACGGCGATGCGGC-3'
IFN-α	5'-TCTCTCCTTTCCTCCTGTCTG-3'	5'-TTCGGTGCAGAATTTGTCTA-3'
IFN-β	5’-TCCCTGAGGAGATTAAGCAG-3’	5’-TCCAGTTTTTCCAGGAC-3’
IFN-γ	5’-ATGAAATATACAAGTTATATCTTGGCT-3’	5’-GCGACAGTTCAGCCATCACTTG-3’
ChIP R1	5´-TAAATAGACCTATTGATTGGAAAGTATGT-3´	5´-GAGAGAGGACAACAGAGTTGTCAG-3´
ChIP R2	5´-TCACCTCTGCACGTCGCATG-3´	5´-ACAGACCAATTTATGCCTACAGCC-3´
ChIP R3	5´-CTACTGTACCTGTCTTTAATCCTGAGTGG-3´	5´-CTGTGTGTAGTTTCTCTCTTATATAGAATG-3´
